# The functions of ocu-miR-205 in regulating hair follicle development in Rex rabbits

**DOI:** 10.1186/s12861-020-00213-5

**Published:** 2020-04-22

**Authors:** Gongyan Liu, Shu Li, Hongli Liu, Yanli Zhu, Liya Bai, Haitao Sun, Shuxia Gao, Wenxue Jiang, Fuchang Li

**Affiliations:** 1 College of Animal Science and Technology, Shandong Agricultural University, Tai’an, 271018 People’s Republic of China; 2 Shandong Provincial Key Laboratory of Animal Biotechnology and Disease Control and Prevention, Tai’an, 271018 People’s Republic of China; 3 Animal Husbandry and Veterinary Institute, Shandong Academy of Agricultural Sciences, Jinan, 251000 People’s Republic of China; 4 Shandong Key Laboratory of Animal Disease Control and Breeding, Jinan, 251000 People’s Republic of China

**Keywords:** Ocu-miR-205, Dermal papilla cell, Rex rabbit, Hair follicle density

## Abstract

**Background:**

Hair follicles are an appendage of the vertebrate epithelium in the skin that arise from the embryonic ectoderm and regenerate cyclically during adulthood. Dermal papilla cells (DPCs) are the key dermal component of the hair follicle that directly regulate hair follicle development, growth and regeneration. According to recent studies, miRNAs play an important role in regulating hair follicle morphogenesis and the proliferation, differentiation and apoptosis of hair follicle stem cells.

**Results:**

The miRNA expression profile of the DPCs from Rex rabbits with different hair densities revealed 240 differentially expressed miRNAs (|log_2_(HD/LD)| > 1.00 and Q-value≤0.001). Among them, ocu-miR-205-5p was expressed at higher levels in DPCs from rabbits with low hair densities (LD) than in rabbits with high hair densities (HD), and it was expressed at high levels in the skin tissue from Rex rabbits (*P* < 0.05). Notably, ocu-miR-205 increased cell proliferation and the cell apoptosis rate, altered the progression of the cell cycle (*P* < 0.05), and modulated the expression of genes involved in the PI3K/Akt, Wnt, Notch and BMP signalling pathways in DPCs and skin tissue from Rex rabbits. It also inhibited the phosphorylation of the CTNNB1 and GSK-3β proteins, decreased the level of the noggin (NOG) protein, and increased the level of phosphorylated Akt (*P* < 0.05). A significant change in the primary follicle density was not observed (*P* > 0.05), but the secondary follicle density and total follicle density (*P* < 0.05) were altered upon interference with ocu-miR-205-5p expression, and the secondary/primary ratio (S/P) in the ocu-miR-205-5p interfered expression group increased 14 days after the injection (*P* < 0.05).

**Conclusions:**

In the present study, ocu-miR-205 promoted the apoptosis of DPCs, altered the expression of genes and proteins involved in the PI3K/Akt, Wnt, Notch and BMP signalling pathways in DPCs and skin from Rex rabbits, promoted the transition of hair follicles from the growth phase to the regression and resting phase, and altered the hair density of Rex rabbits.

## Background

Rex rabbits are typically used for fur production because their fur is short, fine, dense, and smooth and has important economic value [1]. The most important indicator for evaluating the fur quality of Rex rabbit is the density of hair follicles [2]. In recent years, methods to improve the hair follicle density of Rex rabbits have become the most important concern in rabbit production. Hair follicles are an appendage from the vertebrate epithelium in the skin, arise from the embryonic ectoderm, and regenerate cyclically during adulthood [3]. The process of hair follicle formation and differentiation involves at least 20 different cells and tissues [4], including the dermal papilla, hair matrix, inner root sheath, and outer root sheath, as well as different signalling pathways, such as the Wnt, Notch, bone morphogenetic protein (BMP), and fibroblast growth factor (FGF) pathways, among others [5–9]. Phosphatidylinositol 3′-kinase (PI3K) preferentially phosphorylates PIP2 to produce PIP3, and PIP3 is an important second messenger in cells that subsequently activates Akt, and thus it plays an important role in regulating the proliferation and apoptosis of hair follicle cells. The Wnt signalling pathway regulates epithelial morphogenesis, hair follicle development and cell differentiation. The PI3K/Akt signalling pathway inhibits the phosphorylation of β-catenin by phosphorylating glycogen synthase kinase 3β (GSK-3β) and activates the Wnt signalling pathway [5]. Dickkopf-related protein 1 (DKK1) inhibits the Wnt signalling pathway by inhibiting the phosphorylation of β-catenin and induces hair follicle regression [10]. When Notch receptor binds to a ligand, it activates hair follicle stem cells and then promotes the transition of hair follicles from the resting stage to growing stage [6]. The BMP signalling pathway is involved in embryonic skin appendage organ morphogenesis and postnatal hair follicle growth [7]. The BMP2 and BMP4 genes inhibit hair follicle development and are associated with maintaining hair follicles in the resting stage [8]. Noggin (NOG) is an inhibitor of the BMP signalling pathway, and its abnormal expression leads to follicular enlargement [7]. The Notch signalling pathway interacts with the BMP signalling pathway, and the BMP signalling pathway inhibits the Wnt signalling pathway by regulating β-catenin expression [6]. Dermal papilla cells (DPCs) is the key dermal component of the hair follicle, and it can directly regulate hair follicle development, growth and regeneration [11]. Besides, the characteristic of DPCs also affects the size, shape and cycling of hair follicle [12]. Signal exchange between DPCs and hair follicle stem cells at telophase is the key to initiating the next hair follicle cycle [13].

MicroRNAs (miRNAs) are small non-coding RNAs that have been plays an important role in embryogenesis, organ development, cell proliferation, metabolism, embryogenesis, behaviour and other biological processes [14–19]. Recently, miRNAs were reported to play important roles in regulating hair follicle morphogenesis and the proliferation, differentiation and apoptosis of hair follicle stem cells in mice, rats, goats and sheep [20–22]. Notably, miR-214 inhibits hair follicle growth and development by modulating the expression of regulatory factors in Wnt signalling pathway, such as β-catenin and lymphoid enhancer-binding factor 1 (Lef-1) [23]. Upon the overexpression of DKK1 in transgenic mice, the expression of miR-200b and miR-196a in epidermis decreases significantly, which is possibly mediated by potential target genes acting on the Wnt signalling pathway [24]. As shown in a previous study, miR-let-7b promotes alpaca hair growth by inhibiting the transcription of transforming growth factor β receptor 1 (TGFβR1) [25]. BMP4 negatively regulates the expression of miR-21, and miR-21 negatively regulates the expression of the BMP-dependent tumour suppressor genes Pten, Pdcd4, Timp3 and Tpm1 [26]. Hair follicle development and hair growth in mice are regulated by miR-31 through effects on the BMP and Wnt signalling pathways [20]. Additionally, miR-205 is a highly conserved miRNA that shares a similar expression pattern with miR-200 family [27]. It is one of the miRNAs expressed at the highest levels in the epidermis [28, 29], and it plays an essential role in promoting the neonatal expansion of skin stem cells during early development by modulating the PI3K pathway [30].

We isolated the DPCs from the skin of Rex rabbits and analysed the miRNA expression profiles of the DPCs from Rex rabbits with different hair densities to improve the fur quality of Rex rabbits. Among the miRNAs analysed, ocu-miR-205 was one of the miRNAs expressed at the highest levels in DPCs. We examined the function of ocu-miR-205 in hair follicle development.

## Results

### DPCs show a complex miRNA expression pattern

The varieties of miRNAs in DPCs from 30-day-old Rex rabbits with lower and higher hair densities (Additional file 1) were studied by subjecting RNA samples with a high integrity and qualified quality (Additional file 2) to high-throughput small RNA sequencing using the BGISEQ-500 platform. 37,930,744, 39,442,011, 40,965,907, 38,502,653, 40,622,117 and 41,149,163 clean reads were obtained from the six samples (Additional file 3), and the majority of clean reads had a length of 23 nucleotides (Additional file 4). Comparison with known small RNA databases, the percentage of matching reads for each library was 91.91, 91.05, 92.46, 92.77, 90.13, and 91.61%, respectively (Additional file 5). The results of the small RNA classification showed that miRNAs accounted for 80.80, 82.50, 81.60, 85.80, 76.50, and 81.80%, respectively (Additional file 6). The base Q20 value of filtered data was greater than 90% and the Q30 value was greater than 80%. Additional files 7 and 8 show the quantity and quality distribution maps of the bases in each sample, respectively.

**Fig. 1:**
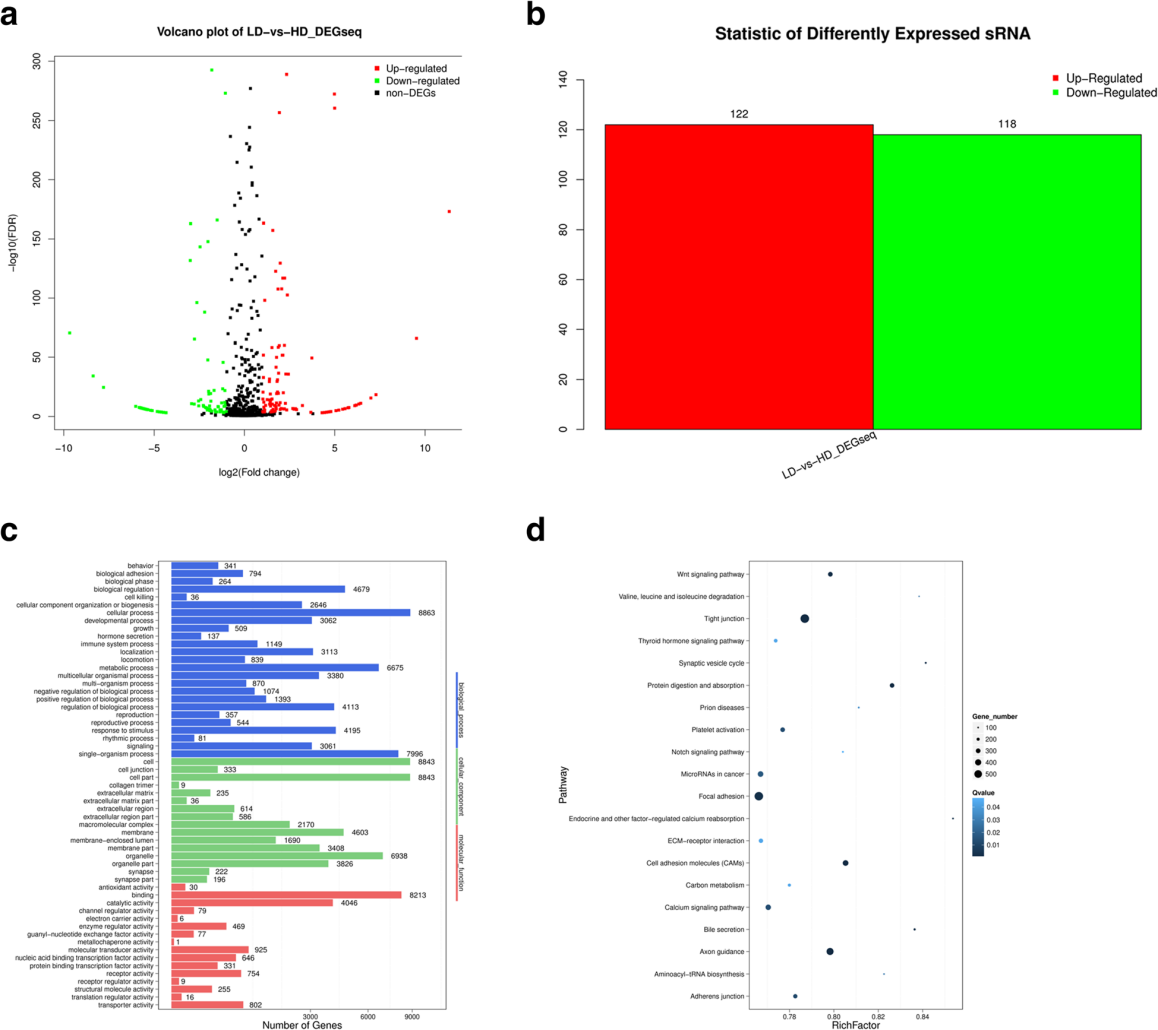
Differentially expressed miRNAs. **a** Differential miRNAs volcanic map; **b** Significantly differential expressed miRNAs; **c** GO functional classification of differential miRNA target genes; **d** Pathway enrichment statistical scatter plot

By screening differentially expressed genes (DEGs), 240 differentially expressed miRNAs were identified (|log_2_(HD/LD)| > 1.00 and Q-value≤0.001; Fig. 1a and Additional file 9), including 122 miRNAs that were upregulated and 118 miRNAs that were downregulated (Fig. 1b). The annotation of the target genes of differentially expressed miRNAs was performed using Gene Ontology (GO) enrichment, and a total of 205,661 target genes were enriched in GO terms (Fig. 1c). Specific GO terms of the target genes mainly involved in the biological process (BP), cellular component (CC) and molecular function (MF) categories. A directed acyclic graph (DAG) of the enriched GO terms in the BP, CC and MF categories is provided in Additional file 10. Following, the target genes were also uploaded into the Kyoto Encyclopaedia of Genes and Genomes (KEGG) database to identify the pathways that were actively regulated by miRNAs in DPCs. Three hundred twenty-five pathways were predicted, and the top enriched pathways were the Wnt signalling pathway and Notch signalling pathway, among others (Fig. 1d).

### The expression of ocu-miR-205

**Fig. 2:**
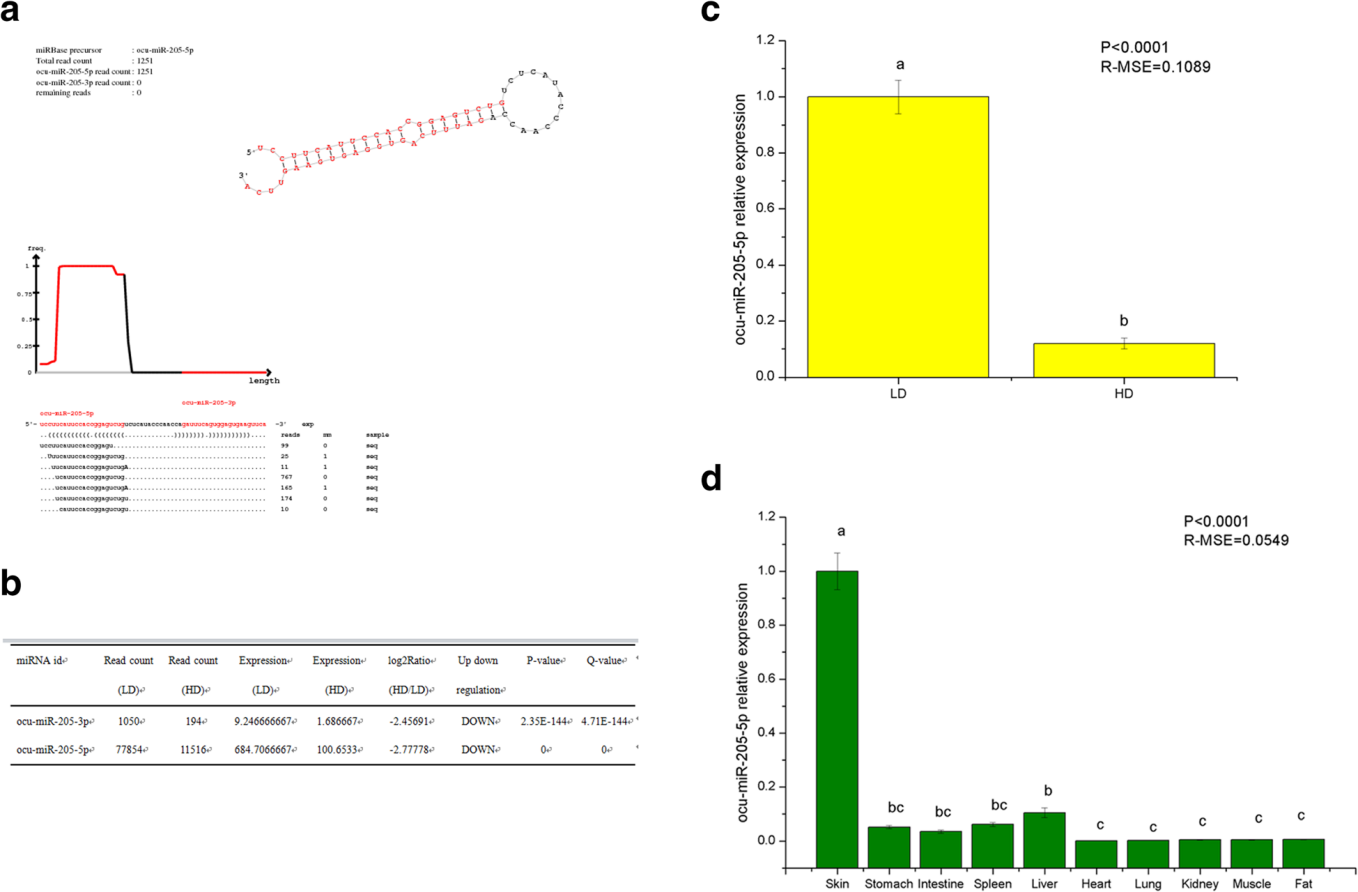
Expression of ocu-miR-205-5p. **a** Structure of ocu-miR-205; **b** Expression of ocu-miR-205 in DPCs of rabbits with different density detected by high-throughput sequencing; **c** Real-time PCR analysis of ocu-miR-205-5p expression in DPCs with different hair density. Mean values ± s.d., *n* = 3, a,b mean *P* < 0.05. **d** Real-time PCR analysis of ocu-miR-205-5p expression in different tissues of Rex rabbits, Mean values ± s.d., *n* = 8, a,b mean *P* < 0.05. Rex rabbits were 30 days old, four for male, four for female

The structure of one of the differentially expressed miRNAs, ocu-miR-205, is shown in Fig. 2a. The expression of ocu-miR-205 differed in DPCs from Rex rabbits with different hair densities (|log_2_(HD/LD)| > 1.00 and Q-value≤0.001; Fig. 2b) and was expressed at low levels in DPCs from Rex rabbits with a high hair density (HD) and at high levels in DPCs from Rex rabbits with a low hair density (LD). Quantitative fluorescence PCR results also confirmed the accuracy of the ocu-miR-205-5p sequencing results (Fig. 2c). Furthermore, the expression of ocu-miR-205-5p in different tissues from Rex rabbits differed (*P* < 0.05). The expression in the skin tissue was higher than in the stomach, intestine, spleen, liver, heart, lung, kidney, muscle and fat (*P* < 0.05; Fig. 2d), suggesting a tissue-specific expression pattern.

### Effects of ocu-miR-205 on DPCs from Rex rabbits

**Fig. 3:**
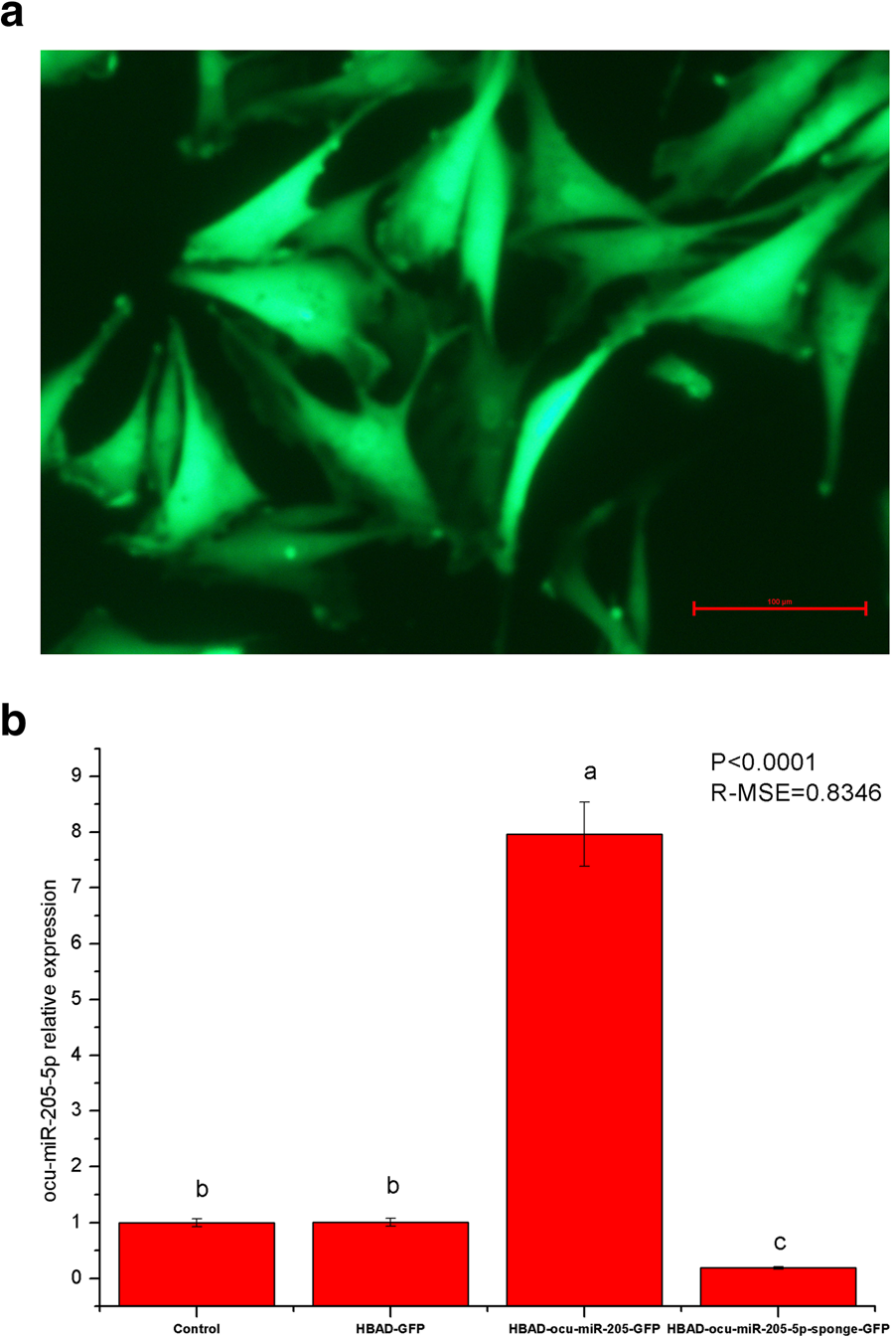
Effects of adenovirus transfection on DPCs. **a** Transfection 24 h, fluorescence microscope (200 magnification); **b** Expression of ocu-miR-205-5p in dermal papilla cells after transfection 48 h detected by quantitative fluorescence quantification, Mean values ± s.d., a,b mean *P* < 0.05, *n* = 8

**Fig. 4:**
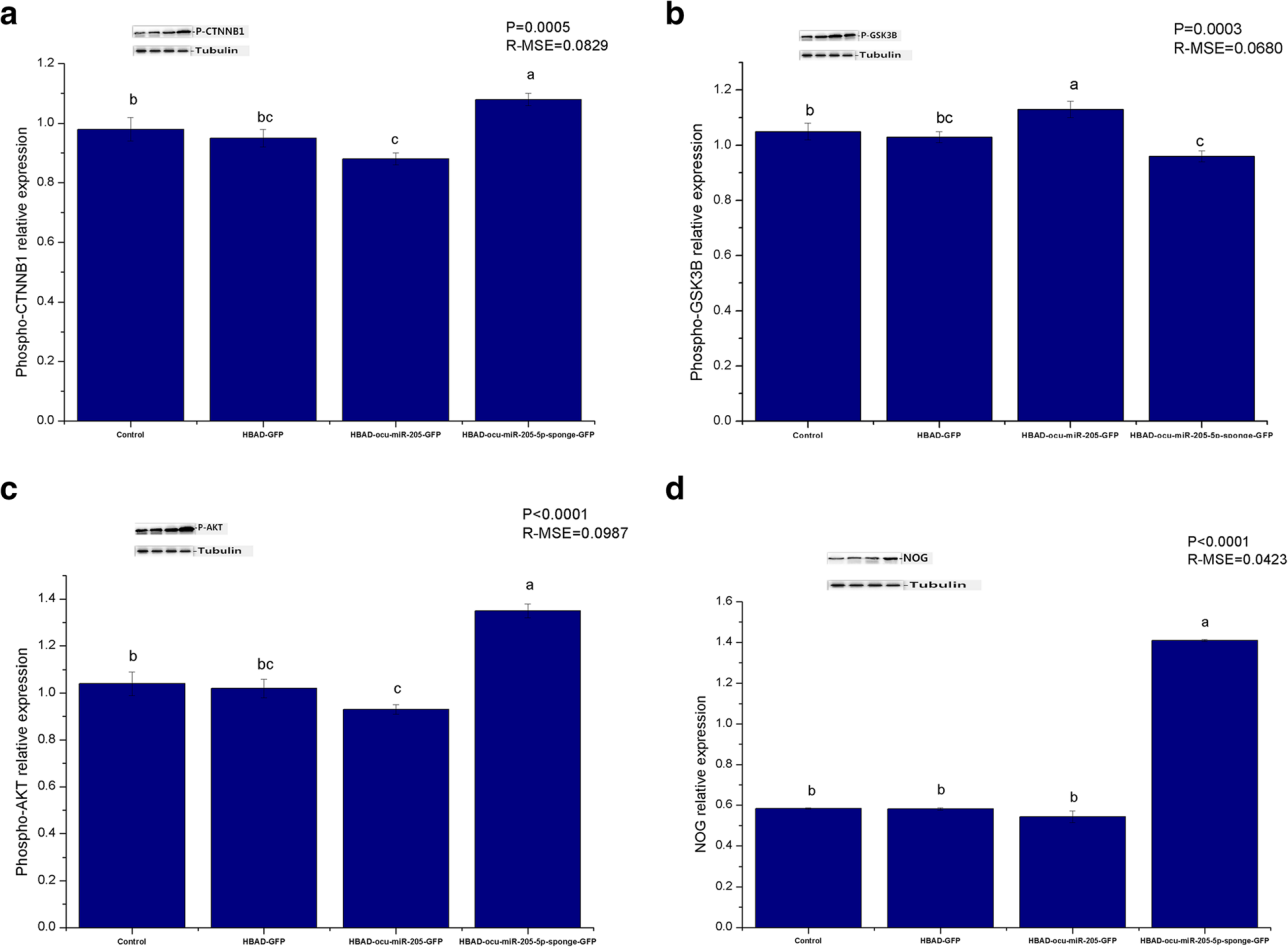
Effects of ocu-miR-205 on expression of hair follicle-related proteins of DPCs. **a** Phospho-CTNNB1; **b** Phospho-GSK-3β; **c** Phospho-Akt; **d** NOG protein. Mean values ±s.d., and *n* = 8 per group. In the same graph, values with same letter superscripts mean no significant difference (*P* > 0.05), with different letter superscripts mean significant difference (*P* < 0.05)

HBAD-GFP, HBAD-ocu-miR-205-GFP, and HBAD-ocu-miR-205-5p-sponge-GFP were constructed and used to transfect DPCs at a multiplicity of infection (MOI) of 200. Cell proliferation, the cell cycle, cell apoptosis, and changes in the expression of ocu-miR-205-5p and hair follicle development-related genes and proteins were detected 48 h after transfection. The constructed adenoviruses successfully transfected DPCs (Fig. 3a), and ocu-miR-205-5p expression increased significantly after overexpression, but decreased significantly after silencing (*P* < 0.05; Fig. 3b). Importantly, ocu-miR-205 increased cell proliferation and the cell apoptosis rate, in addition to altering the progression of the cell cycle (*P* < 0.05; Table 1). Moreover, ocu-miR-205 inhibited the expression of the Inppl1, Frk and Phlda3 mRNAs, which are involved in the PI3K/Akt signalling pathway (*P* < 0.05). The expression of ocu-miR-205 induced the expression of the DKK1 mRNA and inhibited the expression of the Wnt10b, CTNNB1 and GSK-3β genes in the Wnt signalling pathway (*P* < 0.05). The expression of the Notch1, Jagged1, Hes1 and Hes5 genes in the Notch signalling pathway was suppressed by ocu-miR-205 (*P* < 0.05), whereas the expression of the BMP2, BMP4 and TGF-β1 genes in the BMP signalling pathway was induced (*P* < 0.05; Table 2). The expression of ocu-miR-205 inhibited the phosphorylation of the CTNNB1 and GSK-3β proteins, decreased the level of the noggin (NOG) protein, and increased the level of Akt phosphorylation (*P* < 0.05; Fig. 4).

**Table 1:** Effects of ocu-miR-205 on proliferation, cell cycle and apoptosis of dermal papilla cells (DPCs; %)

Items	Group				R-MSE	*P*-value
	Control	HBAD-GFP	HBAD-ocu-miR-205-GFP	HBAD-ocu-miR-205-5p-sponge-GFP		
Proliferation of dermal papilla cells						
Optical density (OD) value	0.56 ± 0.01^b^	0.55 ± 0.01^b^	0.61 ± 0.01^a^	0.34 ± 0.01^c^	0.0356	< 0.0001
Cell cycle of dermal papilla cells						
Resting state/first gap (G0/G1)	87.79 ± 1.00^a^	84.68 ± 1.01^b^	77.71 ± 0.88^c^	85.57 ± 1.03^ab^	2.7795	< 0.0001
Synthesis (S)	7.15 ± 0.85^b^	9.48 ± 0.95^b^	14.73 ± 0.62^a^	8.97 ± 0.68^b^	2.2232	< 0.0001
Second gap/mitosis (G2/M)	5.07 ± 0.24^b^	5.37 ± 0.49^b^	7.56 ± 0.37^a^	5.46 ± 0.35^b^	1.0523	0.0002
Apoptosis of dermal papilla cells						
Early apoptotic ratio (Q4)	33.95 ± 0.40^b^	32.86 ± 1.04^b^	36.71 ± 0.67^a^	29.16 ± 0.47^c^	1.9470	< 0.0001
Later apoptotic ratio (Q2)	33.46 ± 0.57^b^	32.15 ± 1.33^b^	36.11 ± 0.73^a^	31.40 ± 0.57^b^	2.4323	0.0032
Total apoptosis ratio (Q4 + Q2)	67.41 ± 0.51^b^	65.01 ± 1.50^b^	72.83 ± 1.14^a^	60.56 ± 0.47 ^b^	2.8310	< 0.0001

Note: Data shown are mean values ± s. d., and *n* = 8 per group. In the same row, values with same letter superscripts mean no significant difference (*P* > 0.05), with different letter superscripts mean significant difference (*P* < 0.05)

**Table 2:** Effects of ocu-miR-205 on gene expression of signal pathway of dermal papilla cells (DPCs)

Gene	Group				R-MSE	*P*-value
	Control	HBAD-GFP	HBAD-ocu-miR-205-GFP	HBAD-ocu-miR-205-5p-sponge-GFP		
PI3K/Akt signal pathway						
*Inppl1*	1.00 ± 0.09^b^	1.02 ± 0.04^b^	0.80 ± 0.07^b^	1.68 ± 0.12^a^	0.2353	< 0.0001
*Inpp4b*	1.00 ± 0.09	0.97 ± 0.16	0.92 ± 0.08	1.21 ± 0.16	0.3598	0.3966
*Frk*	1.00 ± 0.34^b^	0.90 ± 0.07^b^	0.50 ± 0.13^c^	2.63 ± 0.47^a^	0.8442	0.0001
*Phlda3*	1.00 ± 0.04^b^	0.89 ± 0.06^b^	0.69 ± 0.05^c^	1.18 ± 0.05^a^	0.1491	< 0.0001
Wnt signal pathway						
*Wnt10b*	1.00 ± 0.11^b^	0.95 ± 0.09^b^	0.38 ± 0.11^c^	2.24 ± 0.17^a^	0.3519	< 0.0001
*CTNNB1*	1.00 ± 0.09^b^	1.50 ± 0.13^b^	1.48 ± 0.19^b^	3.16 ± 0.27^a^	0.5186	< 0.0001
*GSK-3β*	1.00 ± 0.14^b^	0.97 ± 0.05^b^	2.66 ± 0.68^a^	3.22 ± 0.71^a^	1.4118	0.0050
*DKK1*	1.00 ± 0.09 ^c^	2.90 ± 0.13^b^	6.09 ± 0.84 ^a^	0.84 ± 0.07 ^c^	1.2141	< 0.0001
Notch signal pathway						
*Notch1*	1.00 ± 0.08^b^	0.96 ± 0.07^b^	0.39 ± 0.02^c^	1.19 ± 0.03^a^	0.1604	< 0.0001
*Jagged1*	1.00 ± 0.07 ^ab^	0.81 ± 0.13 ^bc^	0.60 ± 0.10^c^	1.12 ± 0.07 ^a^	0.2679	0.0032
*Hes1*	1.00 ± 0.12^b^	1.19 ± 0.16^b^	0.90 ± 0.05^b^	2.40 ± 0.33^a^	0.5475	< 0.0001
*Hes5*	1.00 ± 0.16 ^b^	1.04 ± 0.06 ^b^	0.18 ± 0.04^c^	1.38 ± 0.04^a^	0.2477	< 0.0001
BMP signal pathway						
*BMP2*	1.00 ± 0.15^b^	0.51 ± 0.05^bc^	1.69 ± 0.34^a^	0.41 ± 0.05^c^	0.5408	0.0002
*BMP4*	1.00 ± 0.20 ^ab^	1.11 ± 0.18 ^ab^	1.34 ± 0.08^a^	0.63 ± 0.13^b^	0.4405	0.0254
*TGF-β1*	1.00 ± 0.05 ^ab^	0.89 ± 0.18 ^b^	1.33 ± 0.13^a^	0.71 ± 0.07 ^b^	0.3304	0.0071

Note: Data shown are mean values ± s. d., and *n* = 8 per group. In the same row, values with no letter superscripts or same letter superscripts mean no significant difference (*P* > 0.05), with different letter superscripts mean significant difference(*P* < 0.05)

### Effects of ocu-miR-205 on the skin tissue of Rex rabbits

**Fig. 5:**
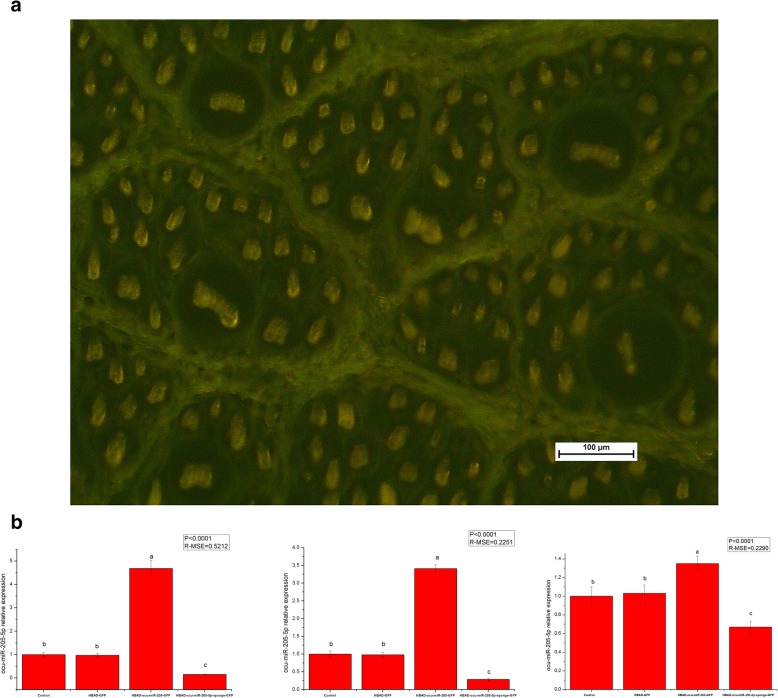
Effects of adenovirus transfection on skin follicles of Rex rabbits. **a** Frozen slice crosscutting of Rex rabbits skin (200 magnification); **b** Expression of ocu-miR-205-5p in dermal papilla cells after transfection 7d, 14d, 21d detected by quantitative fluorescence quantification, Mean values ±s.d., a,b mean *P* < 0.05, *n* = 8

**Fig. 6:**
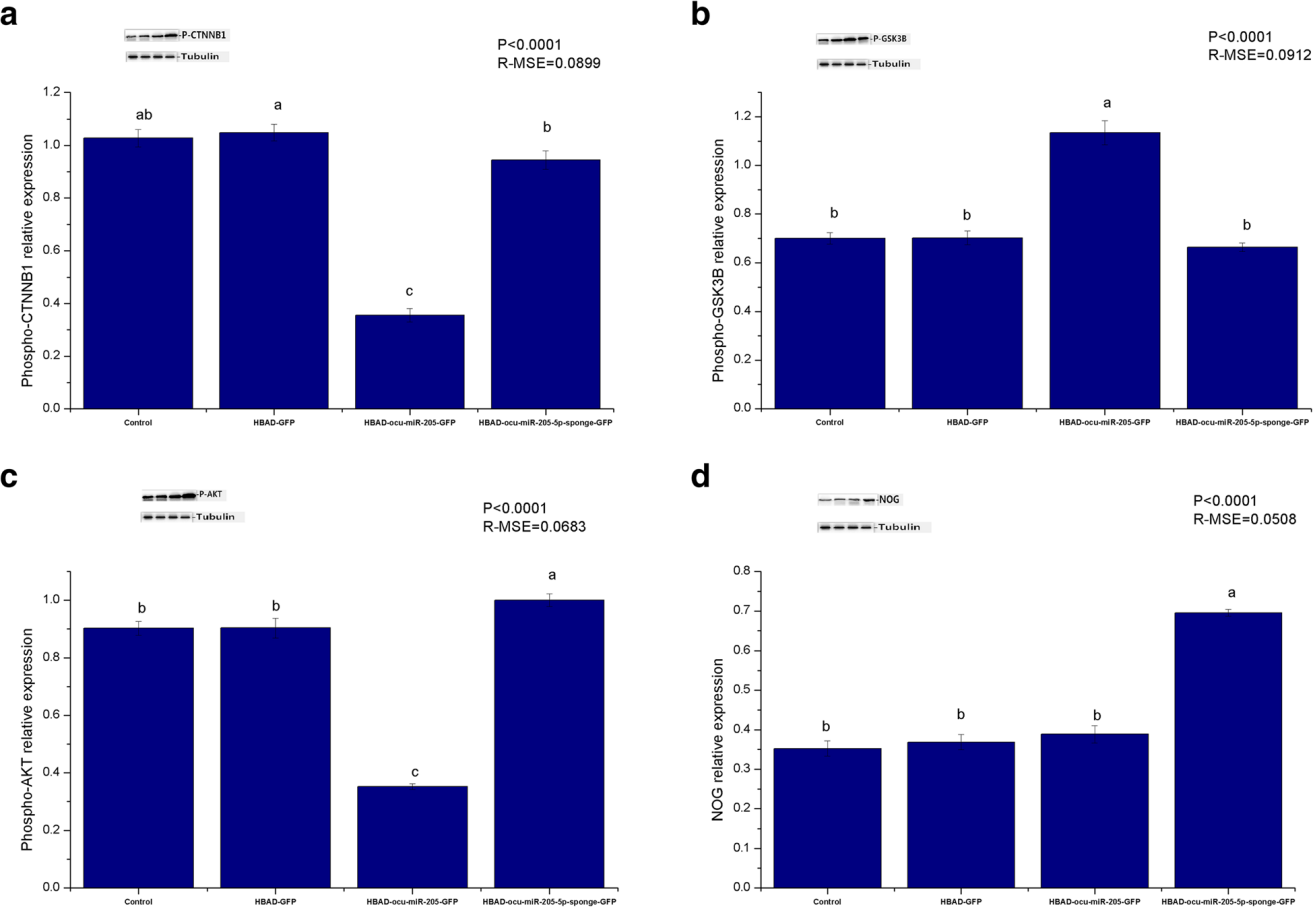
Effects of ocu-miR-205 on expression of hair follicle-related proteins of Rex rabbits skin. **a** Phospho-CTNNB1; **b** Phospho-GSK-3β; **c** Phospho-Akt; **d** NOG protein. Mean values ± s.d., and *n* = 8 per group. In the same graph, values with same letter superscripts mean no significant difference (*P* > 0.05), with different letter superscripts mean significant difference (*P* < 0.05)

One hundred 3-month-old Rex rabbits with similar body weights were randomly divided into 4 groups. After local shaving of the back skin, 50 μL of HBAD-GFP, HBAD-ocu-miR-205-GFP and ocu-miR-205-5p-sponge-GFP were injected intradermally into the rabbits in each group. Twenty-four h after the injected, one randomly selected Rex rabbit from each group was euthanized, and frozen sections were prepared from the locally injected skin to confirm the expression of the miRNAs from the injected adenoviruses. Eight Rex rabbits were randomly selected from each group 7 days, 14 days and 21 days after transfection. After euthanasia, the injected skin was collected and divided into two parts. A part placed in freezing tube and the other part was fixed at a 4% paraformaldehyde fixative solution. The frozen samples were used to detect the changes in the expression of ocu-miR-205-5p and hair follicle development-related genes and proteins. The fixed samples were used to prepare paraffin sections, and the hair density was counted after HE staining. The constructed adenoviruses successfully transfected the skin and hair follicles of Rex rabbits (Fig. 5a), and the expression of ocu-miR-205-5p increased significantly after overexpression, but decreased significantly after silencing (*P* < 0.05; Fig. 5b). Fourteen days after the injection, ocu-miR-205 significantly altered the expression of genes involved in the PI3K/Akt, Wnt, Notch and BMP signalling pathways (*P* < 0.05; Table 3). Moreover, ocu-miR-205 significantly altered the levels of the phosphorylated CTNNB1, GSK-3β, and Akt proteins and the level of the NOG protein (*P* < 0.05; Fig. 6). A significant change in the primary follicle density was not observed (*P* > 0.05), but the secondary follicle density and total follicle density (*P* < 0.05) were altered after ocu-miR-205-5p interfered expression, and the secondary/primary ratio (S/P) in the ocu-miR-205-5p interfered expression group increased at 14 days after the injection (*P* < 0.05; Table 4).

**Table 3:** Effects of ocu-miR-205 on gene expression of signal pathway of Rex rabbits skin

Gene	Group				R-MSE	*P*-value
	Control	HBAD-GFP	HBAD-ocu-miR-205-GFP	HBAD-ocu-miR-205-5p-sponge-GFP		
PI3K/Akt signal pathway						
*Inppl1*	1.00 ± 0.09^b^	0.99 ± 0.07^b^	0.54 ± 0.03^c^	2.30 ± 0.15^a^	0.2731	< 0.0001
*Inpp4b*	1.00 ± 0.15^ab^	0.89 ± 0.07^a^	0.09 ± 0.01^b^	1.05 ± 0.02^a^	0.2348	< 0.0001
*Frk*	1.00 ± 0.26^a^	1.16 ± 0.05^b^	0.31 ± 0.02^c^	1.94 ± 0.20^a^	0.4666	< 0.0001
*Phlda3*	1.00 ± 0.11^b^	1.18 ± 0.11^a^	0.27 ± 0.03^b^	1.18 ± 0.11^a^	0.2500	< 0.0001
Wnt signal pathway						
*Wnt10b*	1.00 ± 0.14^b^	0.86 ± 0.04^bc^	0.74 ± 0.04^c^	1.26 ± 0.09^a^	0.2406	0.0011
*CTNNB1*	1.00 ± 0.07^b^	0.98 ± 0.05^b^	0.75 ± 0.04^c^	1.30 ± 0.08^a^	0.1789	< 0.0001
*GSK-3β*	1.00 ± 0.06^ba^	1.04 ± 0.07^b^	0.85 ± 0.04^b^	1.10 ± 0.05^a^	0.1496	0.0198
*DKK1*	1.00 ± 0.36^a^	0.91 ± 0.10^a^	1.28 ± 0.10^a^	0.18 ± 0.07^b^	0.5625	0.0041
Notch signal pathway						
*Notch1*	1.00 ± 0.18^b^	0.83 ± 0.05^bc^	0.54 ± 0.05^c^	1.95 ± 0.22^a^	0.4131	< 0.0001
*Jagged1*	1.00 ± 0.13^a^	1.02 ± 0.05 ^a^	0.36 ± 0.03^c^	1.34 ± 0.22 ^a^	0.3670	0.0001
*Hes1*	1.00 ± 0.09^b^	1.03 ± 0.06^b^	0.80 ± 0.03^b^	1.49 ± 0.18^a^	0.3042	0.0009
*Hes5*	1.00 ± 0.13 ^b^	1.00 ± 0.05^b^	0.36 ± 0.03^c^	1.43 ± 0.20^a^	0.3435	< 0.0001
BMP signal pathway						
*BMP2*	1.00 ± 0.15^b^	1.02 ± 0.07^b^	1.39 ± 0.08^a^	0.64 ± 0.10^c^	0.3051	0.0005
*BMP4*	1.00 ± 0.15^b^	0.87 ± 0.04^b^	1.58 ± 0.14^a^	0.73 ± 0.05^b^	0.3081	< 0.0001
*TGF-β1*	1.00 ± 0.12 ^ab^	0.88 ± 0.05 ^bc^	1.15 ± 0.06^a^	0.67 ± 0.05^b^	0.2222	0.0014

Note: Data shown are mean values ± s. d., and *n* = 8 per group. In the same row, values with same letter superscripts mean no significant difference (*P* > 0.05), with different letter superscripts mean significant difference (*P* < 0.05)

**Table 4:** Effects of ocu-miR-205 on hair follicle density of Rex rabbits (Count/mm^2^)

Items	Group				R-MSE	*P*-value
	Control	HBAD-GFP	HBAD-ocu-miR-205-GFP	HBAD-ocu-miR-205-5p-sponge-GFP		
Transfection for 7 days						
Hair follicle density	125.57 ± 9.63^bc^	144.25 ± 11.47^ba^	105.02 ± 5.89^c^	163.94 ± 16.68^a^	32.7817	0.0085
Primary hair follicle density	10.08 ± 0.85	9.31 ± 0.85	7.80 ± 0.63	9.79 ± 0.77	2.2046	0.1902
Secondary hair follicle density	115.49 ± 9.48^bc^	134.93 ± 12.01^ba^	97.22 ± 5.68^c^	154.15 ± 16.65^a^	32.9728	0.0113
Secondary/Primary (S/P) ratio	11.92 ± 1.17	16.02 ± 2.67	12.92 ± 1.06	16.38 ± 2.04	5.2469	0.2536
Transfection for 14 days						
Hair follicle density	130.54 ± 12.22^b^	136.62 ± 8.51^b^	119.30 ± 7.24^b^	203.34 ± 14.79^a^	31.4005	< 0.0001
Primary hair follicle density	10.86 ± 0.54	9.96 ± 0.50	11.92 ± 0.74	10.11 ± 0.70	1.7739	0.1307
Secondary hair follicle density	119.68 ± 12.03^b^	126.66 ± 8.28^b^	107.37 ± 7.10^b^	213.32 ± 14.62^a^	30.9035	0.0001
Secondary/Primary (S/P) ratio	11.08 ± 0.96 ^b^	12.82 ± 0.82 ^b^	9.20 ± 0.78 ^b^	21.62 ± 1.94 ^a^	3.4505	< 0.0001
Transfection for 21 days						
Hair follicle density	145.43 ± 13.71^b^	139.58 ± 16.53^b^	126.38 ± 9.30^b^	208.69 ± 21.42^a^	44.8651	0.0049
Primary hair follicle density	14.30 ± 1.19	12.65 ± 0.80	12.92 ± 0.51	15.29 ± 1.28	2.8084	0.2244
Secondary hair follicle density	131.13 ± 13.21^b^	126.94 ± 16.57^b^	113.46 ± 9.27^b^	193.40 ± 21.31^a^	44.4697	0.0060
Secondary/Primary (S/P) ratio	9.45 ± 0.91	10.28 ± 1.40	8.86 ± 0.75	13.28 ± 1.85	3.6808	0.1018

Note: Data shown are mean values ± s. d., and *n* = 8 per group. In the same row, values with no letter superscripts or same letter superscripts mean no significant difference (*P* > 0.05), with different letter superscripts mean significant difference (*P* < 0.05)

## Discussion

Rex rabbits mainly used for fur production, and the fur is the most important livestock products. The hair follicle cycle can typically be divided into anagen, catagen, and telogen phases [31]. The hair follicle of Rex rabbits contains primary and secondary hair follicles, and the different sizes and types of primary and secondary hair follicles is easy distinguished [32]. Besides, the hair follicle growth cycle is easy distinguish the differentiation of hair cycle phases [33]. Many factors affect the growth and development of hair follicles in Rex rabbits, such as genetic, nutritional, age, among others [34–37]. The most hair follicles in anagen phases when the Rex rabbits at 4–5 weeks [37]. Notably, miRNAs plays an important functions in regulating gene expressions [38–40]. Among them, miR-205 is abundantly expressed in the epidermis [28, 29], and it plays an essential role by modulating the PI3K/Akt signalling pathway in promoting the neonatal expansion of skin stem cells during early development [41]. In vitro models, miR-205 promotes keratinocyte migration by targeting the lipid phosphatase SHIP2 and KIR4.1 [42, 43]. There is a potential therapeutic application of DPCs in the treatment of alopecia, because DPCs of various origins induce the de novo formation of the hair follicle structure in both follicular and afollicular epidermis [44–47]. In the present study, ocu-miR-205-5p was expressed at significantly higher levels in DPCs from rabbits with low hair densities than in DPCs from rabbits with high hair densities. Moreover, ocu-miR-205 induced G0/G1 arrest in DPCs, which was further confirmed by the reduction in the population of DPCs in the G0/G1 phase and increase in the apoptotic rate after the transfection of the ocu-miR-205 inhibitor. This finding is consistent with previous studies [48]. Furthermore, overexpression of miR-205 significantly inhibited cell proliferation. Additionally, ocu-miR-205 inhibited the expression of related gene and proteins in the PI3K/Akt, Wnt, and Notch signalling pathways, and activated the BMP signalling pathway. Therefore, ocu-miR-205 plays an important role in regulating hair follicle development.

## Conclusions

In conclusion, ocu-miR-205 promoted the apoptosis of DPCs, inhibited cell proliferation, modulated the expression of genes and proteins involved in the PI3K/Akt, Wnt, Notch and BMP signalling pathways in DPCs and the skin of Rex rabbits, promoted the transition of hair follicles from the growth phase to the regression and resting phase, and altered the hair density of Rex rabbits.

## Methods

### Animal and sample collection

The Rex rabbits used in this study were purchased from Taishan Rabbit Farm (Shandong, China). Ten adult female Rex rabbits with a high (> 14,000/cm^2^, HD) or low hair density (< 10,000/cm^2^, LD) were chosen and divided into 2 groups. An adult male rabbit was selected to mate with the female rabbits within each group. The first generation of offspring (F1 generation) was obtained and then inbred with F1 in the same nest (HD and HD, LD and LD inbreeding). Three rabbits each with high and low hair densities were selected from the second generation of offspring (F2 generation) 30 days after birth. Selected animals were electrically stunned (120 V, pulsed direct current, 50 Hz for 5 s) and euthanized by exsanguination of the carotid artery and before skin was harvested from the experimental rabbits. The DPCs were separated and cultured, methods according to references [49]. After the cells had established a monolayer (approximately 12 days), the total RNA was extracted, tested for quality.

### Construction of the small RNA libraries, sequencing analysis, miRNA identification and prediction of new miRNAs in DPCs

The methods of construction small RNA libraries according to references [50]. Solexa sequencing by synthesis method using the BGISEQ-500 platform at Shenzhen Huada Biotech Co., Ltd. (Shenzhen, China) [51, 52]. The raw reads produced from sequencing were filtered to remove low-quality reads, and the clean reads were analysed using BLAST with Bowtie-1.0.0 software, Rfam [53]. The clean reads were used for miRNA identification and compared with the mature miRNAs and pre-miRNAs from *Oryctolagus cuniculus* listed in miRBase 21.0 [54]. Subsequently, the miRDeep 2 software was used to predict the novel miRNAs by exploring the secondary structure [55], minimum free energy and dicer cleavage sites of the unannotated clean reads that mapped to the *Oryctolagus cuniculus* genome. After the identification of conserved miRNAs, the clean reads were aligned to the *Oryctolagus cuniculus* genome for identify new *Oryctolagus cuniculus* miRNAs.

In order to eliminate the effect of different sequencing quantities on quantitative accuracy, transcripts per million (TPMs) were calculated to standardize the expression levels of small RNA [56]. Based on the assumption that RNA sequencing is a random process, the expression of each transcript is presumed to exhibit a binomial distribution [57]. DEGseq calculated differential expression based on MA-plot [58, 59], and the *P*-value of each miRNA was corrected by performing multiple hypothesis tests using Q-values. When the difference in coincidence was more than two-fold and the Q-value was less than or equal to 0.001, the miRNAs were considered significantly differentially expressed (|log_2_(HD/LD)| > 1.00 and Q-value≤0.001).

The miRNAs were aligned to the EST unigenes of *Oryctolagus cuniculus* and the target genes were predicted using the miRanda algorithm to obtain a better understanding of the potential functions of the significantly differentially expressed miRNAs in Rex rabbits with different hair densities [60]. An enrichment analysis of the predicted target genes was conducted with GO terms and KEGG pathways [61].

### Construction and identification of adenovirus vectors overexpressing and silencing ocu-miR-205

HBAD-GFP (HANBIO adenovirus-green fluorescent protein; empty vector), HBAD-ocu-miR-205-GFP (overexpression), HBAD-ocu-miR-205-5p-sponge-GFP (silencing) adenoviruses were synthesized and constructed by Hanheng Biotechnology Co., Ltd. (Shanghai, China). The infective titres of HBAD-GFP, HBAD-ocu-miR-205-GFP and HBAD-ocu-miR-205-5p-sponge-GFP were 1.26*10^10^ PFU/mL, 1.58*10^10^ PFU/mL and 1.26*10^10^ PFU/mL, respectively.

Third-generation DPCs displaying good growth conditions were inoculated into a disposable 6-well plate. The cell density was approximately 1.0*10^5^ cells/mL. Prior to the infection, the virus was subjected to 10-fold gradient dilution. Generally, the MOI (multiplicity of infection) was controlled in the range of 10–1000. HBAD-GFP, HBAD-ocu-miR-205-GFP and HBAD-ocu-miR-205-GFP were individually transfected into Rex rabbit DPCs at an MOI 200, and a negative control was established using cells undergoing normal culture.

Fifty microliters of the purified adenovirus were injected into the skin of each Rex rabbit with microinjector at a concentration of 5.0*10^8^–1.0 * 10^9^ virus particles per Rex rabbit after shaving the middle part of the back of 100 3-month-old Rex rabbits with similar body weights and good health. Twenty-four h after transfection, one Rex rabbit from each group was randomly selected, euthanized, and frozen sections were prepared from the locally injected skin. The adenovirus-transfected skin was observed under a positive fluorescence microscope (Nikon ECLIPSE 80i, Japan).

### Assessment of the proliferation, cell cycle and apoptosis of DPCs

DPCs were plated in a 96-well plate at a density of 10^4^ cells/well, cultured in basal medium for 24 h, and then transfected with the indicated adenoviruses. The methods assessment of the proliferation according to references [49]. Third-generation DPCs were plated in a disposable 6-well plate at a density of 10^4^ cells/mL, with 2 mL of the cell suspension plated in each well. After a 24-h incubation to allow cells to adhere, the culture medium was removed. After treatment and culture for a certain time, the cells were digested with a trypsin digestion solution lacking EDTA (Solarbio, Beijing, China). The methods assessment of the cell cycle according to references [49]. For apoptosis assessment, cells were centrifuged and collected into a 1.5 mL centrifugal tube, and then washed with PBS. After centrifugation, 500 μL of 10X Annexin V Binding Buffer was added to re-suspended the cells, followed by the labelling of F-actin. Cells were incubated with the FITC Annexin V and Propidium Iodide Staining Solution for 15 min at 4 °C and then analysed using flow cytometry. The percentages of early apoptotic cells (Q4), late apoptotic cells (Q2) and total apoptotic cells (Q2 + Q4) in each sample were calculated.

### Total RNA extraction and real-time PCR analysis

The methods of total RNA extraction and real-time PCR of mRNA was according to references [62]. All quantitative PCR primers were designed using Primer Premier 5 software (Additional file 11). For the quantitative RT-PCR of miRNAs, 1 μg of total RNA was reverse transcribed with Bulge-Loop miRNA-specific reverse transcription primers (RiboBio, China), and quantitative PCR was performed using Fast Start Universal SYBR Green Master Mix (Roche Diagnostics GmbH Mannheim, Germany) and Bulge-Loop primers (RiboBio, Guangzhou, China) on the 7500 Fast System 1.4 system with small nuclear RNA U6 as the normalisation control. The volume of each reaction was 20 μL, including 2 μL of cDNAs, 10 μL of SYBR Green Master (2X), 0.8 μL of the Bulge-Loop™ miRNA Forward Primer (5 μM), 0.8 μL of the Bulge-Loop™ Reverse Primer (5 μM), 0.4 μL of ROX Reference Dye II (50×) and 6.0 μL of ddH_2_O. PCR was performed under the following conditions: 10 min of template denaturation at 95 °C, followed by 40 cycles of 95 °C for 2 s, 60 °C for 20 s, and 70 °C for 10 s. Melting curves (70 °C–95 °C) for each sample were analysed after each run to confirm the specificity of amplification reactions. The relative expression levels of mRNAs and miRNAs were calculated using the arithmetic formula 2^−△△Ct^ [63].

### Western immunoblotting

The methods of total protein extraction and SDS-PAGE were according to references [62]. The membranes were blocked with 5% skimmed milk in phosphate-buffered saline (PBS; Solarbio, China) at 4 °C overnight and incubated with primary antibodies (tubulin AT819, Beyotime, China; phospho-CTNNB1-S552 pAb, Abcam, US; phospho-GSK3B-S9 pAb, Abcam, US; phospho-AKT1-S473 pAb, Abcam, US; or NOG polyclonal antibody, Abcam, US). The membranes were then rinsed with Tris-buffered saline containing Tween (TBST; Solarbio, China), and subjected to detection with a 1:3000 dilution of a horseradish peroxidase (HRP)-conjugated goat anti-mouse IgG antibody (Beyotime, China) at 37 °C for 1 h. Proteins were visualized using BeyoECL reagents (Beyotime, China). The intensity of the bands was quantified with a Pro Plus 6.0 Biological Image Analysis System. The levels of phospho-CTNNB1, phospho-GSK3B, phospho-AKT1 and NOG were normalized to the internal control beta-tubulin, and the relative expression levels were calculated.

### Statistical analysis

All data were analysed with SAS software (SAS version 8e; SAS Institute, Cary, NC, USA). A one-way ANOVA was used to evaluate the differences in mean values among various groups. The data are presented as the means and R-MSE. *P* < 0.05 was regarded as statistically significant.

## Supplementary information

**Additional file 1: Supplementary Figure 1.** Skin crosscutting HE staining of Rex rabbits with different hair density (100 magnification)(a) Low hair density (b) High hair density. **Supplementary Table 1.** Statistics of hair follicle numbers of Rex rabbits with different hair density. Note: Data shown are mean values ± s. d., and *n* = 3 per group. In the same row, values with with different letter superscripts mean significant difference(*P* < 0.05).

**Additional file 2: Supplementary Table 2.** RNA quality of six DPC samples. **Supplementary Fig. 2.** RNA quality (a) Agarose electrophoresis of RNA in DPCs; (b) Quality test results of total RNA by Agilent 2100.

**Additional file 3: Supplementary Table 3.** Statistics of sequence data of each samples.

**Additional file 4: Supplementary Figure 3.** Length distribution map of Small RNA. The X-axis is the length of small RNA, and the Y-axis is the corresponding number of small RNA.

**Additional file 5: Supplementary Table 4.** Alignment clean tag in genome.

**Additional file 6: Supplementary Figure 4.** Statistical distribution map of small RNA types. In order to make each unique small RNA have a unique annotation, the annotation statistics of small RNA traverse the annotation according to the priority order of miRNA > piRNA > snoRNA > Rfam > other sRNA.

**Additional file 7: Supplementary Figure 5.** The quantity distribution maps of clean tag base in each sample. The X-axis is the position of the base in read, and the Y-axis represents the proportion of the base.

**Additional file 8: Supplementary Figure 6.** The quality distribution maps of the clean tag base in each sample. The X-axis is the position of base in read, and the Y-axis represents the base mass value.

**Additional file 9: Supplementary Table 5.** LD-vs-HD_DEGseq. diffexpfilter.

**Additional file 10: Supplementary Figure 7.** Directed acyclic graph of GO enrichment. (a) LD-vs-HD_DEGseq. Biological_Process. Top GO; LD-vs-HD_DEGseq. Cellular_Component. Top GO; LD-vs-HD_DEGseq. Molecular_Function. Top GO. The branches in the figure represent the inclusion relationship, and the function range defined from top to bottom is getting smaller and smaller. The box represents the top 5 GO terms of each classification enrichment degree, and through the inclusion relationship, the associated GO terms are displayed together. The name of the term and the *P*-value corrected by enrichment analysis are displayed on each node. The darker the color (red) indicates the smaller the P-value and the higher the enrichment degree.

**Additional file 11: Supplementary Table 6.** Primer sequence information in experiments.

## Data Availability

Small RNA sequencing data has been deposited in Sequence Read Archive of the National Center for Biotechnology Information under temporary submission ID: SUB7193686, and BioSample accessions: SAMN14476334, SAMN14476335, SAMN14476336, SAMN14476337, SAMN14476338, SAMN14476339.

## Abbreviations

BMP: Bone morphogenetic protein
BP: Biological process
CC: Cellular component
DAG: Directed acyclic graph
DEGs: Differentially expressed genes
DPCs: Dermal papilla cells
DKK1: Dickkopf-related protein 1
FGF: Fibroblast growth factor
GAPDH: Glyceraldehyde-3-phosphate dehydrogenase
GFP: Green fluorescent protein
GO: Gene Ontology
GSK-3β: Glycogen synthase kinase 3β
HD: High hair density
HE: Haematoxylin and eosin
Lef-1: Lymphoid enhancer-binding factor 1
LD: Low hair density
miRNAs: MicroRNAs
mRNA: Messenger RNA
NOG: Noggin
S/P: Secondary/primary ratio
SDS: Sodium dodecyl sulfate
TGFβ: Transforming growth factor β
TPM: Transcripts per million
ocu-miR-205: *Oryctolagus cuniculus* microRNA 205
PAGE: Polyacrylamide gel electrophoresis
PBS: Phosphate-buffered saline
PI3K: Phosphatidylinositol 3′-kinase
