# Supplementary material for: The functions of ocu-miR-205 in regulating hair follicle development in Rex rabbits
Source: BMC Dev Biol. 2020 Apr 22;20:8. doi: 10.1186/s12861-020-00213-5 (PMC7178635; doi:10.1186/s12861-020-00213-5)
Supplement: Supplementary file 1 — Additional file 1: Supplementary Figure 1. Skin crosscutting HE staining of Rex rabbits with different hair density (100 magnification)(a) Low hair density (b) High hair density. Supplementary Table 1. Statistics of hair follicle numbers of Rex rabbits with different hair density. Note: Data shown are mean values ± s. d., and n = 3 per group. In the same row, values with with different letter superscripts mean significant difference(P < 0.05). [file 12861_2020_213_MOESM1_ESM.zip › Supplementary Table 1.docx]

Supplementary Table 1. Statistics of hair follicle numbers of Rex rabbits with different hair density

| Items | Different hair follicle density | | R-MSE | *P*-value |
| --- | --- | --- | --- | --- |
|  | Low density (LD) | High density (HD) |  |  |
| Hair follicle density (Count/mm^2^) | 152.00±6.11^b^ | 182.67±2.96 ^a^ | 8.3166 | 0.0107 |

Note: Data shown are mean values ± s. d., and n =3 per group. In the same row, values with with different letter superscripts mean significant difference(*P*<0.05).
