# Supplementary material for: The functions of ocu-miR-205 in regulating hair follicle development in Rex rabbits
Source: BMC Dev Biol. 2020 Apr 22;20:8. doi: 10.1186/s12861-020-00213-5 (PMC7178635; doi:10.1186/s12861-020-00213-5)
Supplement: Supplementary file 2 — Additional file 2: Supplementary Table 2. RNA quality of six DPC samples. Supplementary Fig. 2. RNA quality (a) Agarose electrophoresis of RNA in DPCs; (b) Quality test results of total RNA by Agilent 2100. [file 12861_2020_213_MOESM2_ESM.zip › Supplementary Table 2.docx]

Supplementary Table 2. RNA quality of six dermal papilla cell samples

| Sample name | Concentration (ng/ μL) | Volume (μL) | Total (μg) | RIA | 28S/18S |
| --- | --- | --- | --- | --- | --- |
| LD1 | 568 | 25 | 14.20 | 10.0 | 2.2 |
| LD2 | 567 | 25 | 14.18 | 10.0 | 2.1 |
| LD3 | 385 | 25 | 9.63 | 10.0 | 2.0 |
| HD1 | 508 | 25 | 12.70 | 10.0 | 2.1 |
| HD2 | 360 | 25 | 9.00 | 10.0 | 1.9 |
| HD3 | 462 | 25 | 11.55 | 10.0 | 2.2 |
