# Supplementary material for: The functions of ocu-miR-205 in regulating hair follicle development in Rex rabbits
Source: BMC Dev Biol. 2020 Apr 22;20:8. doi: 10.1186/s12861-020-00213-5 (PMC7178635; doi:10.1186/s12861-020-00213-5)
Supplement: Supplementary file 3 — Additional file 3: Supplementary Table 3. Statistics of sequence data of each samples. [file 12861_2020_213_MOESM3_ESM.docx]

Supplementary Table 3. Statistics of sequence data of each sample

| Items | Sample name | | | | | |
| --- | --- | --- | --- | --- | --- | --- |
|  | LD1 | LD2 | LD3 | HD1 | HD2 | HD3 |
| Total raw reads | 41780505 | 41931741 | 43766730 | 41075486 | 43457108 | 43799741 |
| Low quality tag count | 634583 | 514665 | 345688 | 495739 | 484764 | 466868 |
| Invalid adapter tag count | 1648457 | 1480626 | 1400707 | 1412236 | 1849500 | 1309939 |
| PolyA tag count | 1023 | 403 | 748 | 510 | 578 | 583 |
| Short valid length tag | 1565698 | 494036 | 1053680 | 664348 | 500149 | 873188 |
| Total clean reads | 37930744 | 39442011 | 40965907 | 38502653 | 40622117 | 41149163 |
| Clean reads ratio (%) | 90.79 | 94.06 | 93.60 | 93.74 | 93.48 | 93.95 |
| Q20 of clean tag (%) | 99.20 | 99.20 | 99.20 | 99.10 | 99.20 | 99.00 |
| Q30 of clean tag (%) | 95.15 | 95.18 | 95.26 | 95.25 | 95.22 | 95.16 |
