# Supplementary figures and images for: The functions of ocu-miR-205 in regulating hair follicle development in Rex rabbits
Source: BMC Dev Biol. 2020 Apr 22;20:8. doi: 10.1186/s12861-020-00213-5 (PMC7178635; doi:10.1186/s12861-020-00213-5)

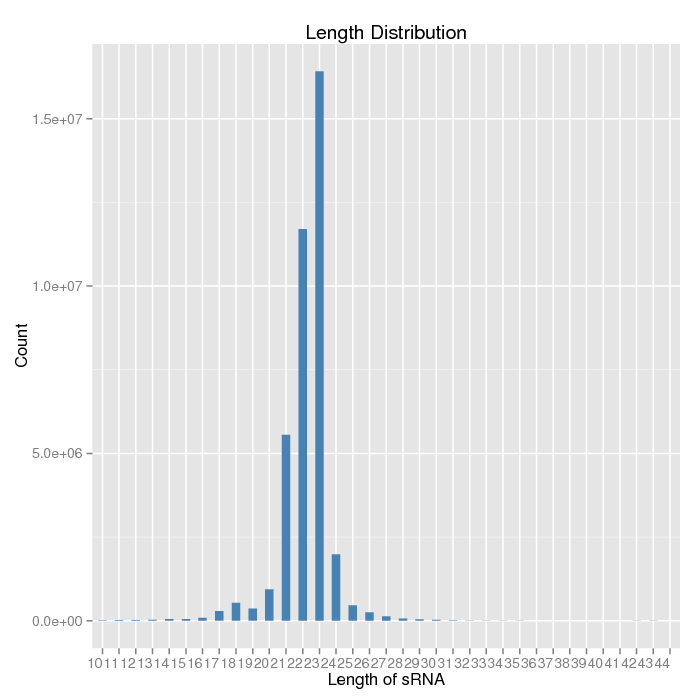

Supplement: Supplementary file 4 — Additional file 4: Supplementary Figure 3. Length distribution map of Small RNA. The X-axis is the length of small RNA, and the Y-axis is the corresponding number of small RNA. [file 12861_2020_213_MOESM4_ESM.zip › Supplementary Figure 3 HD1.png]

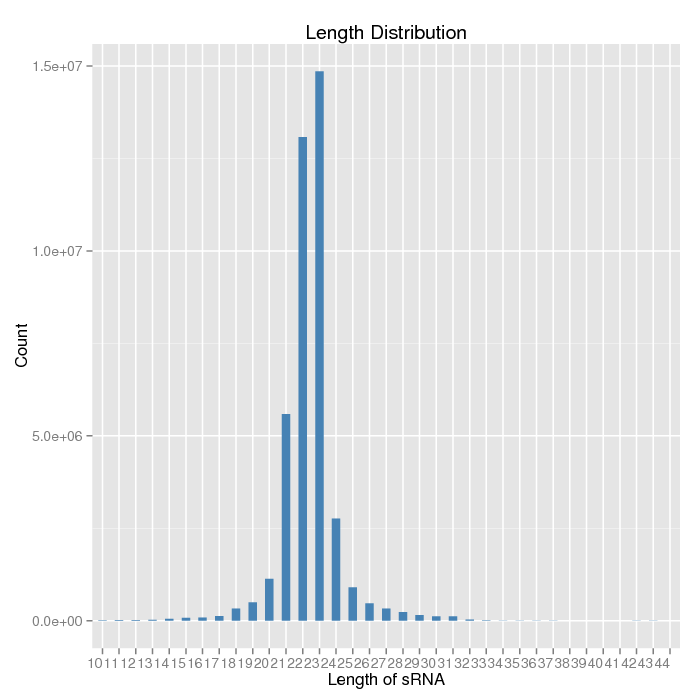

Supplement: Supplementary file 4 — Additional file 4: Supplementary Figure 3. Length distribution map of Small RNA. The X-axis is the length of small RNA, and the Y-axis is the corresponding number of small RNA. [file 12861_2020_213_MOESM4_ESM.zip › Supplementary Figure 3 HD2.png]

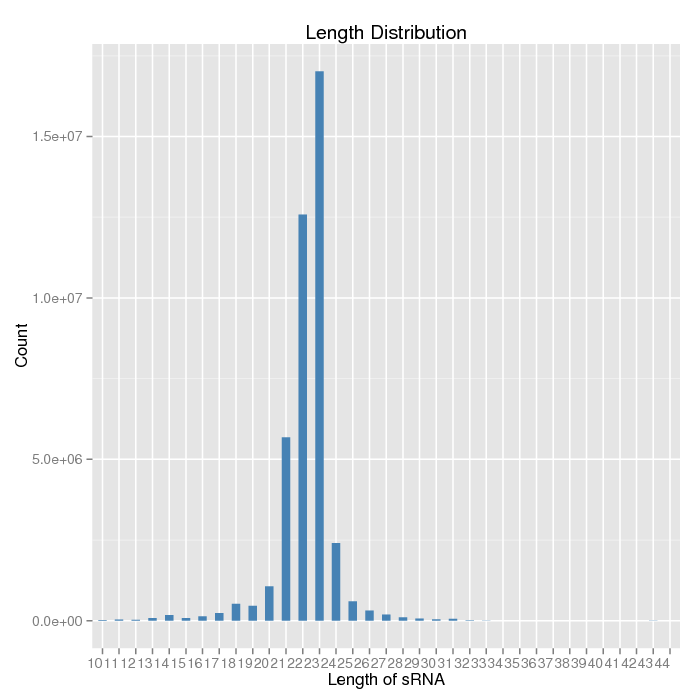

Supplement: Supplementary file 4 — Additional file 4: Supplementary Figure 3. Length distribution map of Small RNA. The X-axis is the length of small RNA, and the Y-axis is the corresponding number of small RNA. [file 12861_2020_213_MOESM4_ESM.zip › Supplementary Figure 3 HD3.png]

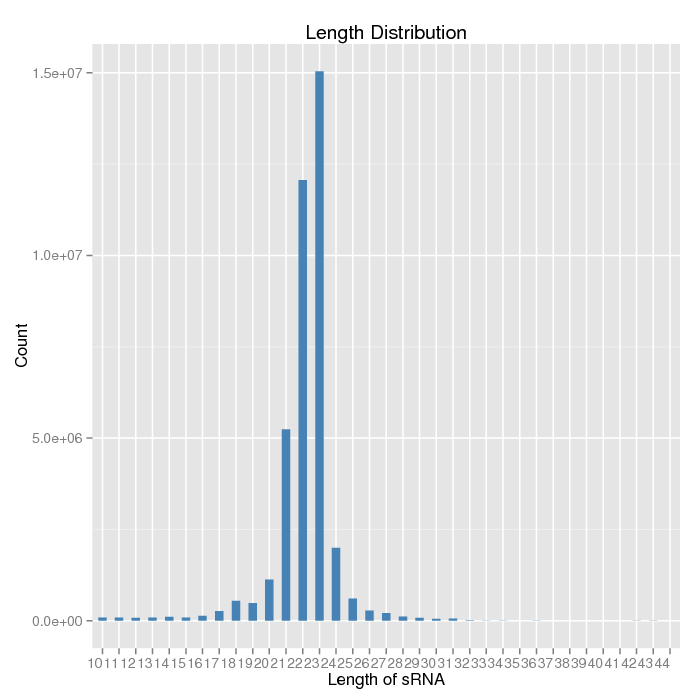

Supplement: Supplementary file 4 — Additional file 4: Supplementary Figure 3. Length distribution map of Small RNA. The X-axis is the length of small RNA, and the Y-axis is the corresponding number of small RNA. [file 12861_2020_213_MOESM4_ESM.zip › Supplementary Figure 3 LD1.png]

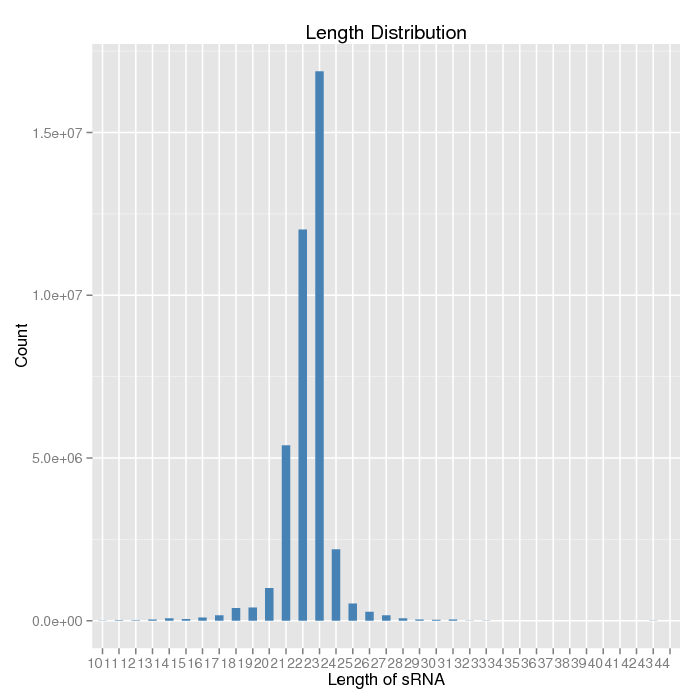

Supplement: Supplementary file 4 — Additional file 4: Supplementary Figure 3. Length distribution map of Small RNA. The X-axis is the length of small RNA, and the Y-axis is the corresponding number of small RNA. [file 12861_2020_213_MOESM4_ESM.zip › Supplementary Figure 3 LD2.png]

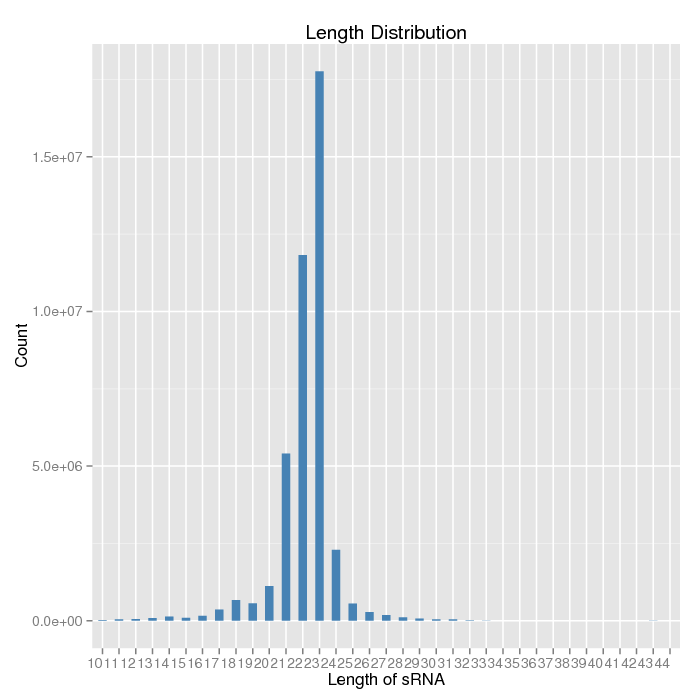

Supplement: Supplementary file 4 — Additional file 4: Supplementary Figure 3. Length distribution map of Small RNA. The X-axis is the length of small RNA, and the Y-axis is the corresponding number of small RNA. [file 12861_2020_213_MOESM4_ESM.zip › Supplementary Figure 3 LD3.png]

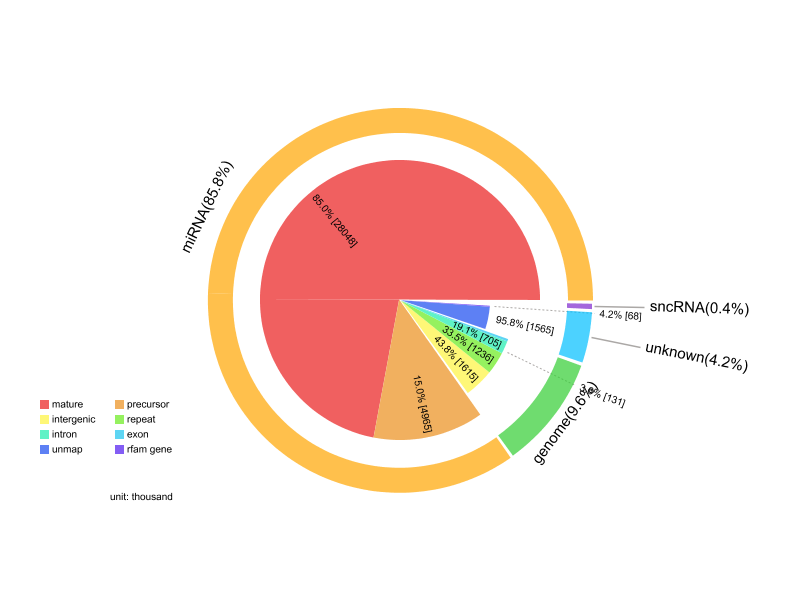

Supplement: Supplementary file 6 — Additional file 6: Supplementary Figure 4. Statistical distribution map of small RNA types. In order to make each unique small RNA have a unique annotation, the annotation statistics of small RNA traverse the annotation according to the priority order of miRNA > piRNA > snoRNA > Rfam > other sRNA. [file 12861_2020_213_MOESM6_ESM.zip › Supplementary Figure 4 HD1.png]

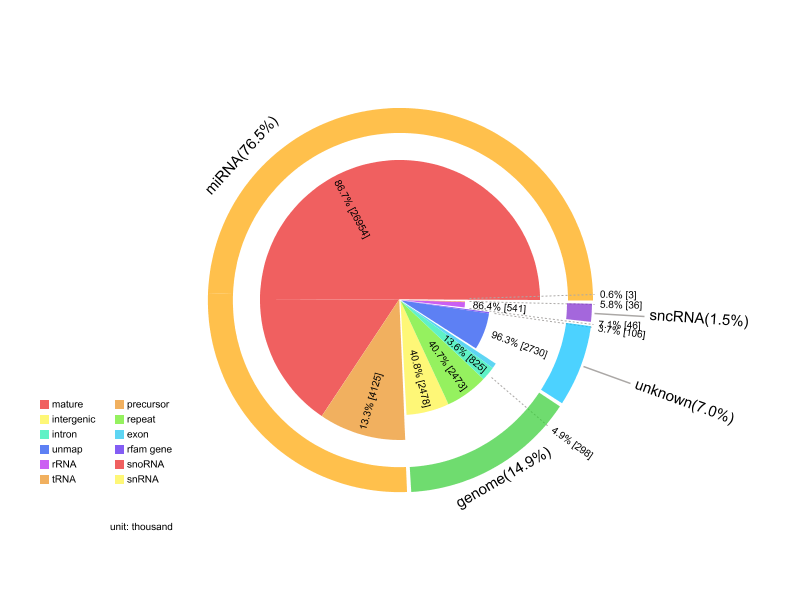

Supplement: Supplementary file 6 — Additional file 6: Supplementary Figure 4. Statistical distribution map of small RNA types. In order to make each unique small RNA have a unique annotation, the annotation statistics of small RNA traverse the annotation according to the priority order of miRNA > piRNA > snoRNA > Rfam > other sRNA. [file 12861_2020_213_MOESM6_ESM.zip › Supplementary Figure 4 HD2.png]

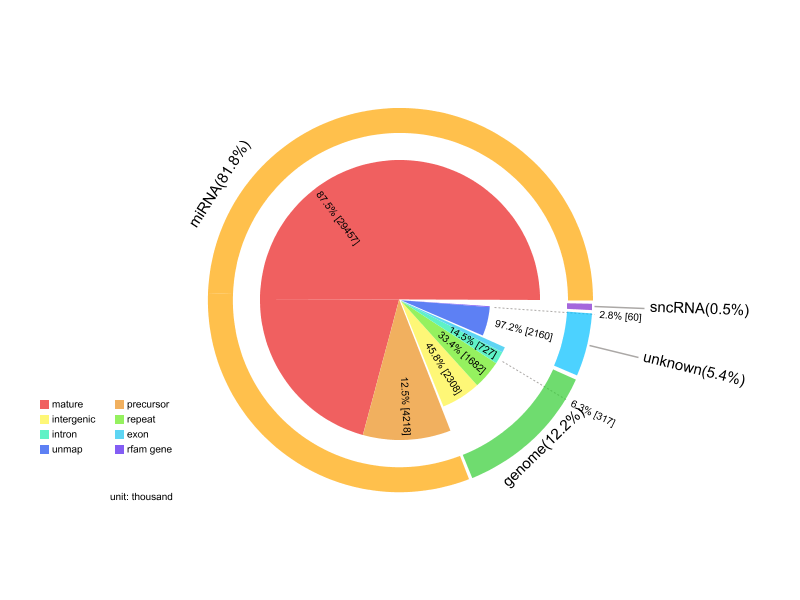

Supplement: Supplementary file 6 — Additional file 6: Supplementary Figure 4. Statistical distribution map of small RNA types. In order to make each unique small RNA have a unique annotation, the annotation statistics of small RNA traverse the annotation according to the priority order of miRNA > piRNA > snoRNA > Rfam > other sRNA. [file 12861_2020_213_MOESM6_ESM.zip › Supplementary Figure 4 HD3.png]

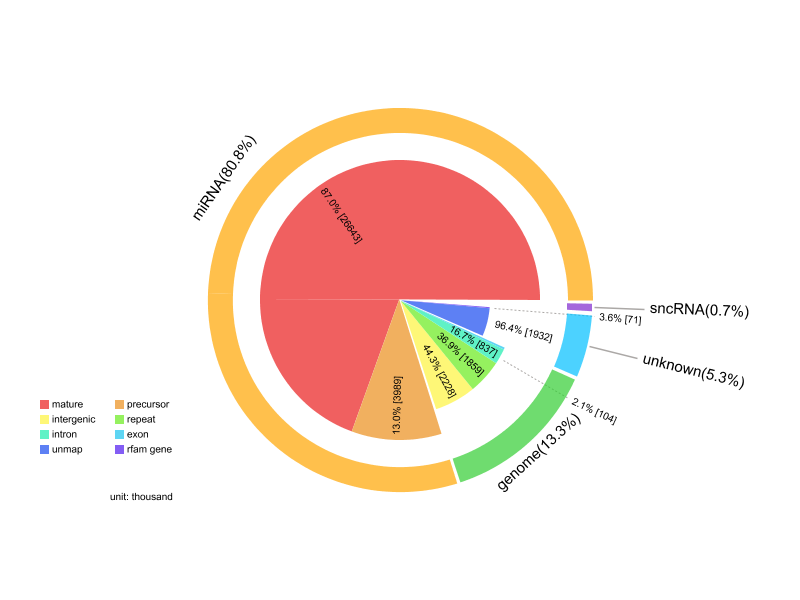

Supplement: Supplementary file 6 — Additional file 6: Supplementary Figure 4. Statistical distribution map of small RNA types. In order to make each unique small RNA have a unique annotation, the annotation statistics of small RNA traverse the annotation according to the priority order of miRNA > piRNA > snoRNA > Rfam > other sRNA. [file 12861_2020_213_MOESM6_ESM.zip › Supplementary Figure 4 LD1.png]

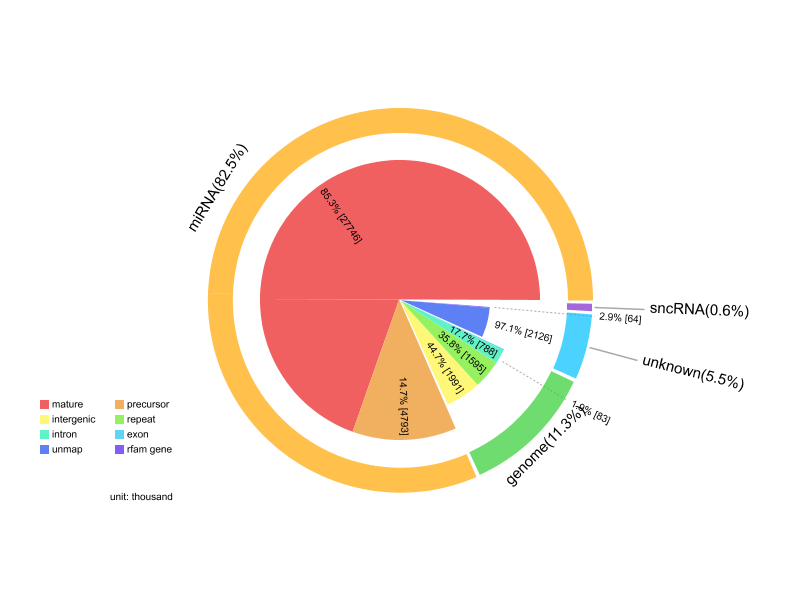

Supplement: Supplementary file 6 — Additional file 6: Supplementary Figure 4. Statistical distribution map of small RNA types. In order to make each unique small RNA have a unique annotation, the annotation statistics of small RNA traverse the annotation according to the priority order of miRNA > piRNA > snoRNA > Rfam > other sRNA. [file 12861_2020_213_MOESM6_ESM.zip › Supplementary Figure 4 LD2.png]

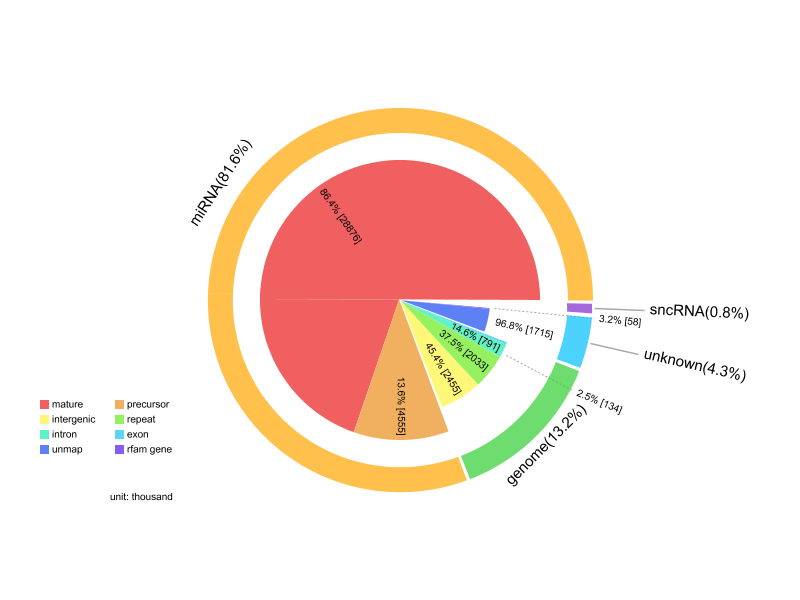

Supplement: Supplementary file 6 — Additional file 6: Supplementary Figure 4. Statistical distribution map of small RNA types. In order to make each unique small RNA have a unique annotation, the annotation statistics of small RNA traverse the annotation according to the priority order of miRNA > piRNA > snoRNA > Rfam > other sRNA. [file 12861_2020_213_MOESM6_ESM.zip › Supplementary Figure 4 LD3.png]

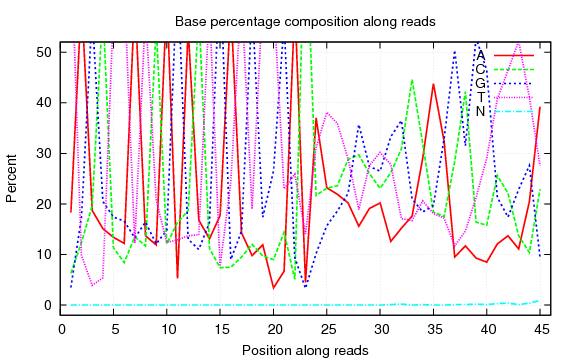

Supplement: Supplementary file 7 — Additional file 7: Supplementary Figure 5. The quantity distribution maps of clean tag base in each sample. The X-axis is the position of the base in read, and the Y-axis represents the proportion of the base. [file 12861_2020_213_MOESM7_ESM.zip › Supplementary Figure 5 HD1.png]

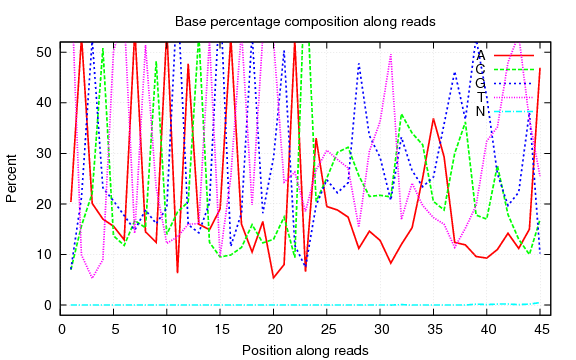

Supplement: Supplementary file 7 — Additional file 7: Supplementary Figure 5. The quantity distribution maps of clean tag base in each sample. The X-axis is the position of the base in read, and the Y-axis represents the proportion of the base. [file 12861_2020_213_MOESM7_ESM.zip › Supplementary Figure 5 HD2.png]

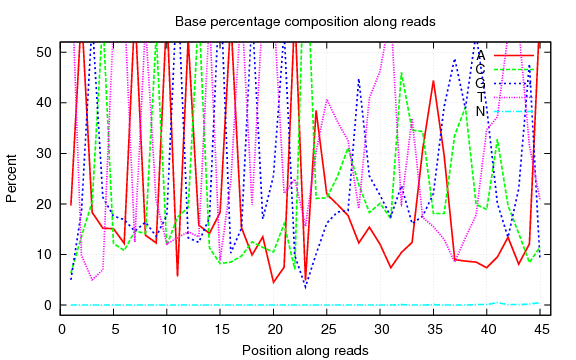

Supplement: Supplementary file 7 — Additional file 7: Supplementary Figure 5. The quantity distribution maps of clean tag base in each sample. The X-axis is the position of the base in read, and the Y-axis represents the proportion of the base. [file 12861_2020_213_MOESM7_ESM.zip › Supplementary Figure 5 HD3.png]

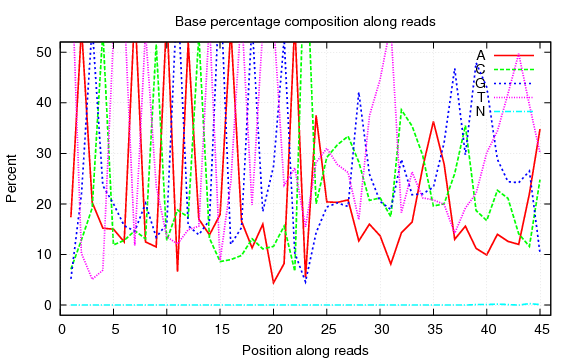

Supplement: Supplementary file 7 — Additional file 7: Supplementary Figure 5. The quantity distribution maps of clean tag base in each sample. The X-axis is the position of the base in read, and the Y-axis represents the proportion of the base. [file 12861_2020_213_MOESM7_ESM.zip › Supplementary Figure 5 LD1.png]

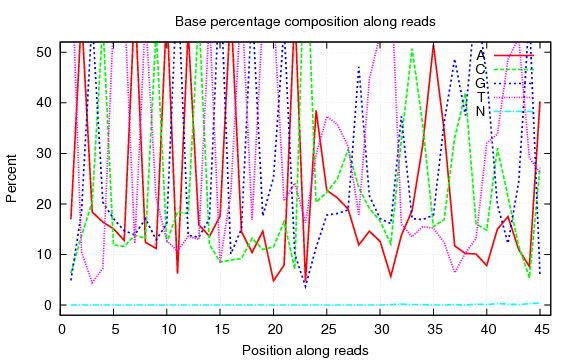

Supplement: Supplementary file 7 — Additional file 7: Supplementary Figure 5. The quantity distribution maps of clean tag base in each sample. The X-axis is the position of the base in read, and the Y-axis represents the proportion of the base. [file 12861_2020_213_MOESM7_ESM.zip › Supplementary Figure 5 LD2.png]

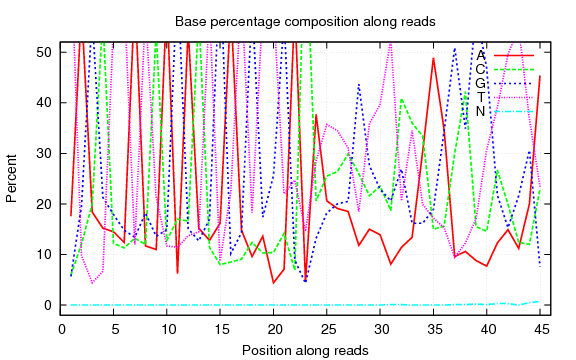

Supplement: Supplementary file 7 — Additional file 7: Supplementary Figure 5. The quantity distribution maps of clean tag base in each sample. The X-axis is the position of the base in read, and the Y-axis represents the proportion of the base. [file 12861_2020_213_MOESM7_ESM.zip › Supplementary Figure 5 LD3.png]

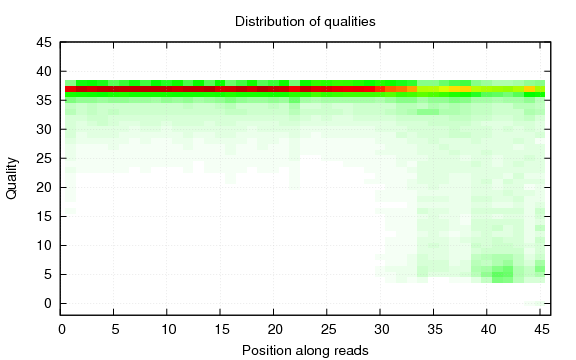

Supplement: Supplementary file 8 — Additional file 8: Supplementary Figure 6. The quality distribution maps of the clean tag base in each sample. The X-axis is the position of base in read, and the Y-axis represents the base mass value. [file 12861_2020_213_MOESM8_ESM.zip › Supplementary Figure 6 HD1.png]

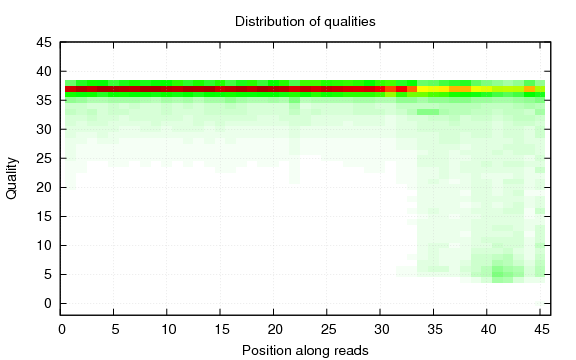

Supplement: Supplementary file 8 — Additional file 8: Supplementary Figure 6. The quality distribution maps of the clean tag base in each sample. The X-axis is the position of base in read, and the Y-axis represents the base mass value. [file 12861_2020_213_MOESM8_ESM.zip › Supplementary Figure 6 HD2.png]

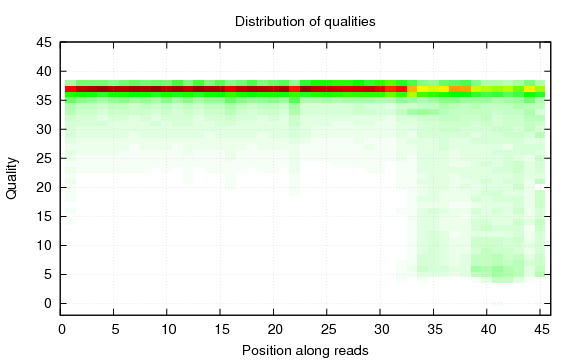

Supplement: Supplementary file 8 — Additional file 8: Supplementary Figure 6. The quality distribution maps of the clean tag base in each sample. The X-axis is the position of base in read, and the Y-axis represents the base mass value. [file 12861_2020_213_MOESM8_ESM.zip › Supplementary Figure 6 HD3.png]

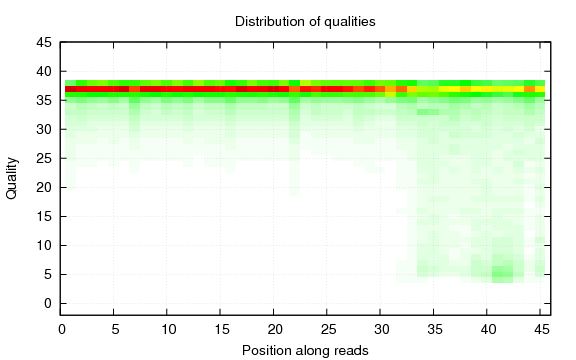

Supplement: Supplementary file 8 — Additional file 8: Supplementary Figure 6. The quality distribution maps of the clean tag base in each sample. The X-axis is the position of base in read, and the Y-axis represents the base mass value. [file 12861_2020_213_MOESM8_ESM.zip › Supplementary Figure 6 LD1.png]

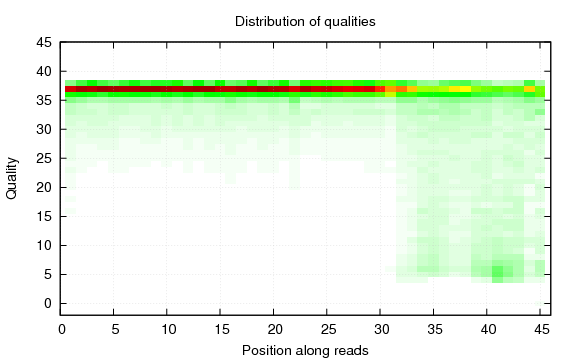

Supplement: Supplementary file 8 — Additional file 8: Supplementary Figure 6. The quality distribution maps of the clean tag base in each sample. The X-axis is the position of base in read, and the Y-axis represents the base mass value. [file 12861_2020_213_MOESM8_ESM.zip › Supplementary Figure 6 LD2.png]

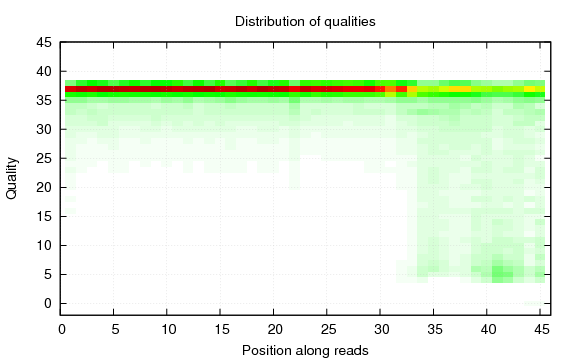

Supplement: Supplementary file 8 — Additional file 8: Supplementary Figure 6. The quality distribution maps of the clean tag base in each sample. The X-axis is the position of base in read, and the Y-axis represents the base mass value. [file 12861_2020_213_MOESM8_ESM.zip › Supplementary Figure 6 LD3.png]

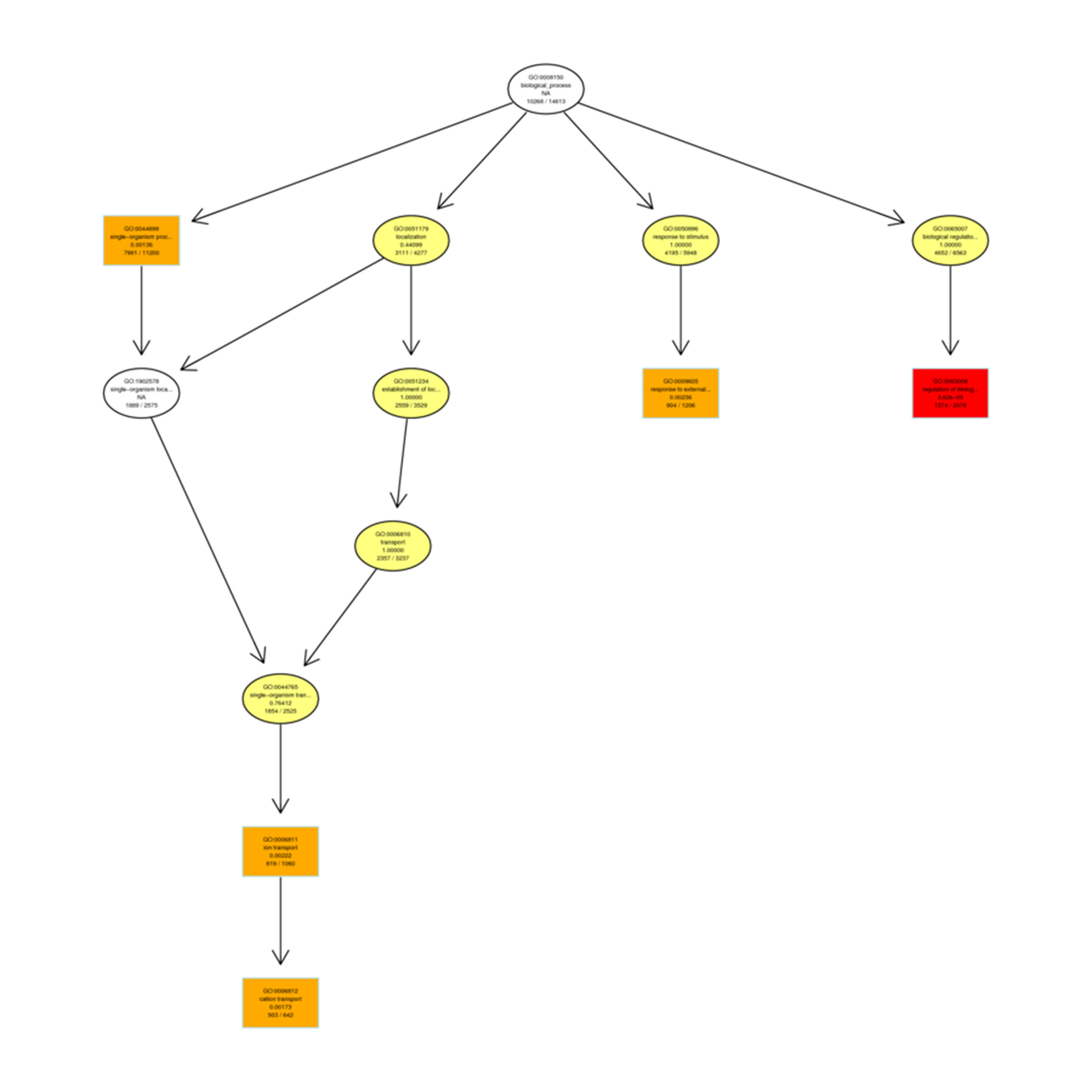

Supplement: Supplementary file 10 — Additional file 10: Supplementary Figure 7. Directed acyclic graph of GO enrichment. (a) LD-vs-HD_DEGseq. Biological_Process. Top GO; LD-vs-HD_DEGseq. Cellular_Component. Top GO; LD-vs-HD_DEGseq. Molecular_Function. Top GO. The branches in the figure represent the inclusion relationship, and the function range defined from top to bottom is getting smaller and smaller. The box represents the top 5 GO terms of each classification enrichment degree, and through the inclusion relationship, the associated GO terms are displayed together. The name of the term and the P-value corrected by enrichment analysis are displayed on each node. The darker the color (red) indicates the smaller the P-value and the higher the enrichment degree. [file 12861_2020_213_MOESM10_ESM.zip › Supplementary Figure 7a.jpg]

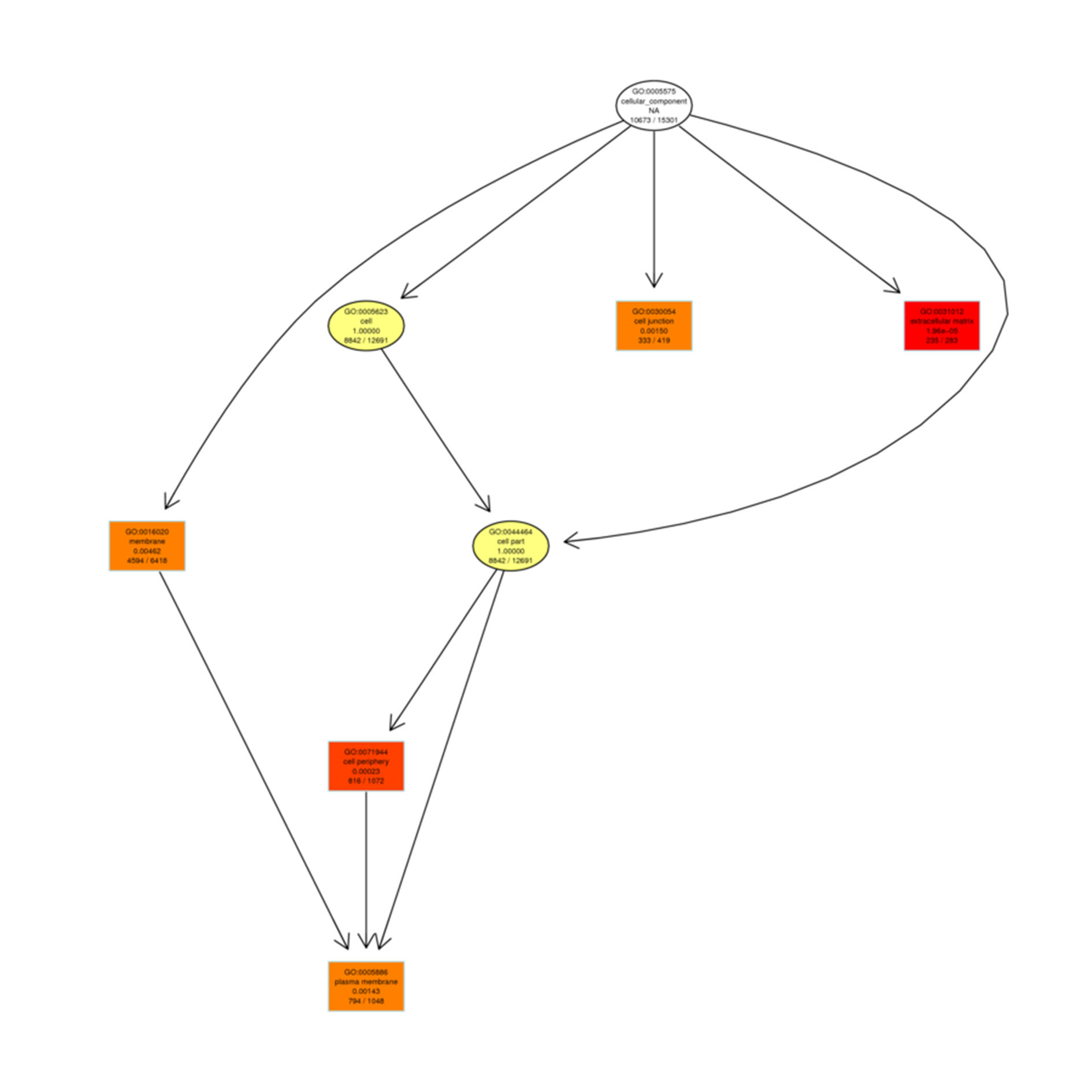

Supplement: Supplementary file 10 — Additional file 10: Supplementary Figure 7. Directed acyclic graph of GO enrichment. (a) LD-vs-HD_DEGseq. Biological_Process. Top GO; LD-vs-HD_DEGseq. Cellular_Component. Top GO; LD-vs-HD_DEGseq. Molecular_Function. Top GO. The branches in the figure represent the inclusion relationship, and the function range defined from top to bottom is getting smaller and smaller. The box represents the top 5 GO terms of each classification enrichment degree, and through the inclusion relationship, the associated GO terms are displayed together. The name of the term and the P-value corrected by enrichment analysis are displayed on each node. The darker the color (red) indicates the smaller the P-value and the higher the enrichment degree. [file 12861_2020_213_MOESM10_ESM.zip › Supplementary Figure 7b.jpg]

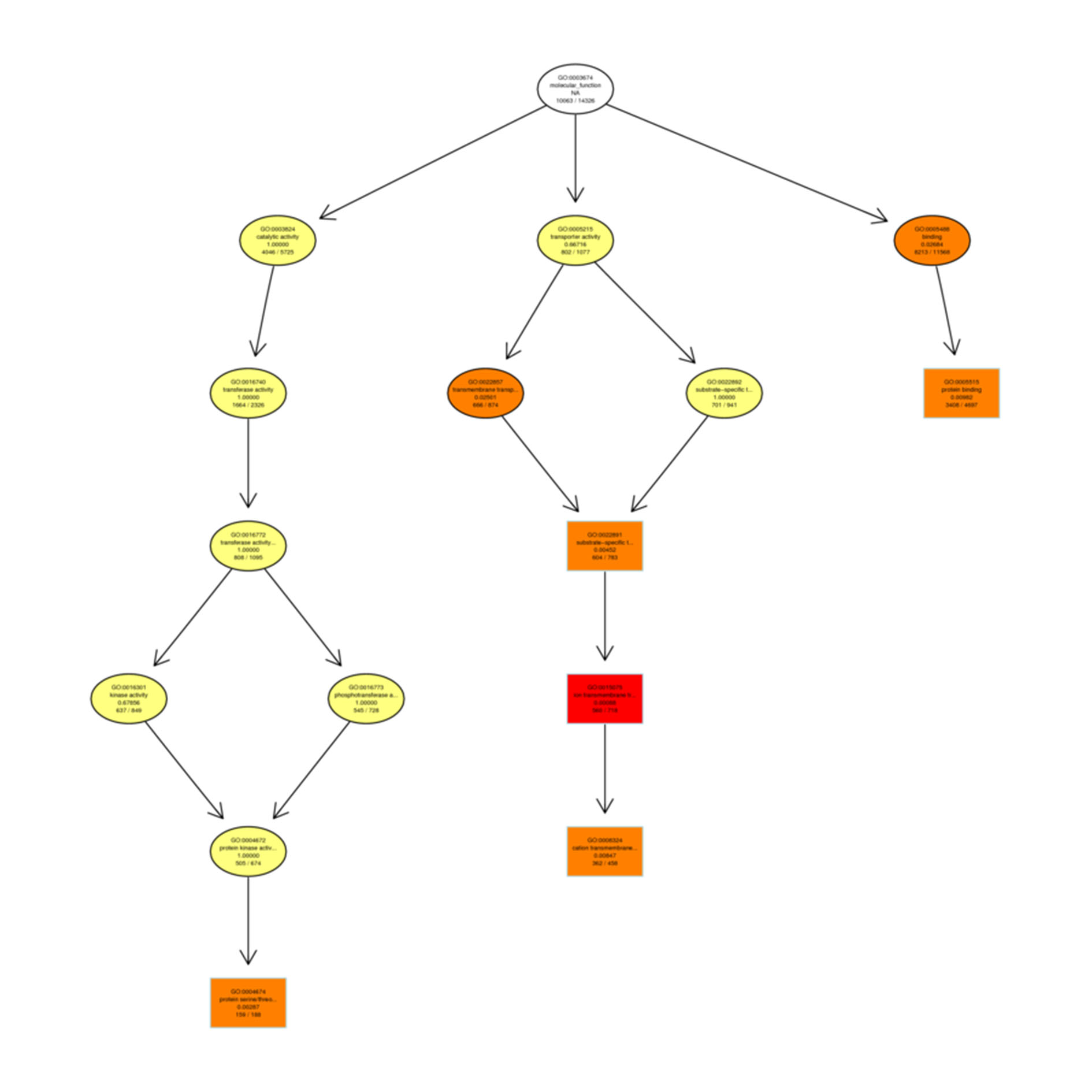

Supplement: Supplementary file 10 — Additional file 10: Supplementary Figure 7. Directed acyclic graph of GO enrichment. (a) LD-vs-HD_DEGseq. Biological_Process. Top GO; LD-vs-HD_DEGseq. Cellular_Component. Top GO; LD-vs-HD_DEGseq. Molecular_Function. Top GO. The branches in the figure represent the inclusion relationship, and the function range defined from top to bottom is getting smaller and smaller. The box represents the top 5 GO terms of each classification enrichment degree, and through the inclusion relationship, the associated GO terms are displayed together. The name of the term and the P-value corrected by enrichment analysis are displayed on each node. The darker the color (red) indicates the smaller the P-value and the higher the enrichment degree. [file 12861_2020_213_MOESM10_ESM.zip › Supplementary Figure 7c.jpg]
