# Supplementary material for: The functions of ocu-miR-205 in regulating hair follicle development in Rex rabbits
Source: BMC Dev Biol. 2020 Apr 22;20:8. doi: 10.1186/s12861-020-00213-5 (PMC7178635; doi:10.1186/s12861-020-00213-5)
Supplement: Supplementary file 5 — Additional file 5: Supplementary Table 4. Alignment clean tag in genome. [file 12861_2020_213_MOESM5_ESM.docx]

Supplementary Table 4. Alignment clean tag in genome

| Sample name | Total tag | Mapped tag | Percentage (%) |
| --- | --- | --- | --- |
| LD1 | 37930744 | 34863360 | 91.91 |
| LD2 | 39442011 | 35910940 | 91.05 |
| LD3 | 40965907 | 37877015 | 92.46 |
| HD1 | 38502653 | 35719844 | 92.77 |
| HD2 | 40622117 | 36613764 | 90.13 |
| HD3 | 41149163 | 37698053 | 91.61 |

#### 
