# Supplementary material for: The functions of ocu-miR-205 in regulating hair follicle development in Rex rabbits
Source: BMC Dev Biol. 2020 Apr 22;20:8. doi: 10.1186/s12861-020-00213-5 (PMC7178635; doi:10.1186/s12861-020-00213-5)
Supplement: Supplementary file 11 — Additional file 11: Supplementary Table 6. Primer sequence information in experiments. [file 12861_2020_213_MOESM11_ESM.docx]

Supplementary Table 6 Primer sequence information in experiments

| Gene names | Genbank accession number | Specific primers | Product size (bp) | |
| --- | --- | --- | --- | --- |
| GAPDH | NM_001082253 | F:5’-TGCCACCCACTCCTCTACCTTCG-3’ | 118 | |
|  |  | R:5’-CGAAGGTAGGGATGGGTGGCA-3’ |  | |
| Inppl1 | NM_017349797 | F:5’-CAGAGCACGAGAACCGCATCAG-3’ | 199 | |
|  |  | R:5’-AGCCGCAGGATGTCCAGGTAG-3’ |  | |
| Inpp4b | NM_008267416 | F: 5’-TCCTAAGAGCACAGCGGAGAGC -3’ | 195 | |
|  |  | R:5’-GCTGCCTTCACTGCCACCATC -3’ |  | |
| Frk | XM_008263392 | F:5’- CAAGCGATGGCCTCTGTGTCAG-3’ | 195 | |
|  |  | R:5’- CTGCTACTGGAGTGGTGTTGTTCC -3’ |  | |
| Phlda3 | XM_002717603 | F: 5’-GCGGCGGCGAGATTGACTTC -3’ | 101 | |
|  |  | R:5’-CTGGATGGCCTGCTGGTTCTTG -3’ |  | |
| Wnt10b | NM_002711076 | F:5’-TGTGCCATCCCTCTTCCTTA-3’ | 150 | |
|  |  | R:5’-GGCTCCACCTCTAACTTCTGC-3’ |  | |
| CTNNB1 | DQ786777 | F: 5’-TTCTTGGGACTCTTGTTCAGC-3’ | 122 | |
|  |  | R:5’-CACTTGGCACACCATCATCT-3’ |  | |
| GSK-3β | NM_001146156 | F: 5’-ATCCATGTCTCCCTGTCCAC-3’ | 119 | |
|  |  | R:5’-TTTCCTCTTCCCACTCCTGA-3’ |  | |
| DKK_1_ | NM_001082737 | F: 5’-ATTCCAACGCCATCAAGAAC-3’ | 163 | |
|  |  | R:5’-CCACACTCCTCGTCCTCTGT-3’ |  | |
| Notch1 | XM_011518717 | F:5’- TGCGAGACCAACATCAACGAGTG-3’ | 94 | |
|  |  | R:5’- TCAGGCAGAAGCAGAGGTAGGC-3’ |  | |
| Jagged1 | XM_018261778 | F: 5’- TGGAGGAGGACGACATGGACAAG -3’ | 176 | |
|  |  | R:5’- CATCCGATTGAGGCTCTGTGCTG-3’ |  | |
| Hes1 | XM_002716517 | F: 5’- CCAGATCAACGCCATGACCTATCC-3’ | 200 | |
|  |  | R:5’- ACACCTTAGCCGCCTCTCCAG-3’ |  | |
| Hes5 | NM_008253710 | F: 5’- AGACCGCATCAACAGCAGCATC-3’ | 105 | |
|  |  | R:5’- ATCTCCAGGATGTCCGCCTTCTC-3’ |  | |
| BMP2 | XM_001082650 | F: 5’- GACATCCTGAGCGAGTTCGAGTTG-3’ | 113 | |
|  |  | R:5’- CGGCGGTACAAGTCCAGCATG-3’ |  | |
| BMP4 | NM_001195723 | F: 5’- CTAAGCATCACCCACAGCGG-3’ | 163 | |
|  |  | R:5’- CAGTCATTCCAGCCCACGTC-3’ |  | |
| TGFβ-1 | NM_008249704 | F: 5’- CCGTTTCTTTCGTGGGATAC-3’ | | 108 |
|  |  | R:5’-GGTAAGGGAGGAGGGTCTCA-3’ | |  |
